# Amino Acid Starvation-Induced Glutamine Accumulation Enhances Pneumococcal Survival

**DOI:** 10.1128/msphere.00625-22

**Published:** 2023-04-05

**Authors:** Chengwang Zhang, Yanhong Liu, Haoran An, Xueying Wang, Lina Xu, Haiteng Deng, Songquan Wu, Jing-Ren Zhang, Xiaohui Liu

**Affiliations:** a Department of Basic Medical Science, School of Medicine, Lishui University, Lishui, Zhejiang, China; b Center for Infectious Disease Research, Department of Basic Medical Science, School of Medicine, Tsinghua University, Beijing, China; c Tsinghua-Peking Center for Life Sciences, Tsinghua University, Beijing, China; d National Protein Science Facility, Tsinghua University, Beijing, China; e School of Life Sciences, Tsinghua University, Beijing, China; University of Nebraska Medical Center

**Keywords:** *Streptococcus pneumoniae*, methionine starvation, glutamine accumulation, cellular pH, survival, intracellular pH, metabolome, methionine

## Abstract

Bacteria are known to cope with amino acid starvation by the stringent response signaling system, which is mediated by the accumulation of the (p)ppGpp alarmones when uncharged tRNAs stall at the ribosomal A site. While a number of metabolic processes have been shown to be regulatory targets of the stringent response in many bacteria, the global impact of amino acid starvation on bacterial metabolism remains obscure. This work reports the metabolomic profiling of the human pathogen Streptococcus pneumoniae under methionine starvation. Methionine limitation led to the massive overhaul of the pneumococcal metabolome. In particular, methionine-starved pneumococci showed a massive accumulation of many metabolites such as glutamine, glutamic acid, lactate, and cyclic AMP (cAMP). In the meantime, methionine-starved pneumococci showed a lower intracellular pH and prolonged survival. Isotope tracing revealed that pneumococci depend predominantly on amino acid uptake to replenish intracellular glutamine but cannot convert glutamine to methionine. Further genetic and biochemical analyses strongly suggested that glutamine is involved in the formation of a “prosurvival” metabolic state by maintaining an appropriate intracellular pH, which is accomplished by the enzymatic release of ammonia from glutamine. Methionine starvation-induced intracellular pH reduction and glutamine accumulation also occurred to various extents under the limitation of other amino acids. These findings have uncovered a new metabolic mechanism of bacterial adaptation to amino acid limitation and perhaps other stresses, which may be used as a potential therapeutic target for infection control.

**IMPORTANCE** Bacteria are known to cope with amino acid starvation by halting growth and prolonging survival via the stringent response signaling system. Previous investigations have allowed us to understand how the stringent response regulates many aspects of macromolecule synthesis and catabolism, but how amino acid starvation promotes bacterial survival at the metabolic level remains largely unclear. This paper reports our systematic profiling of the methionine starvation-induced metabolome in S. pneumoniae. To the best of our knowledge, this represents the first reported bacterial metabolome under amino acid starvation. These data have revealed that the significant accumulation of glutamine and lactate enables S. pneumoniae to form a “prosurvival” metabolic state with a lower intracellular pH, which inhibits bacterial growth for prolonged survival. Our findings have provided insightful information on the metabolic mechanisms of pneumococcal adaptation to nutrient limitation during the colonization of the human upper airway.

## INTRODUCTION

Adaptation to shortages of amino acids and other nutrients is vital for bacterial survival and evolution. Escherichia coli and many other bacteria detect amino acid starvation by the ribosome-associated RelA/SpoT homologue (RSH) enzymes, leading to growth arrest and prolonged survival (1). When uncharged tRNAs stall at the ribosomal A site, the RSH enzymes activate the stringent response signaling system by synthesizing the alarmones guanosine 5′-monophosphate 3′-diphosphate (pGpp), guanosine tetraphosphate (ppGpp), and guanosine pentaphosphate (pppGpp), collectively referred to as (pp)pGpp (2–4). (pp)pGpp signaling nucleotides broadly regulate many aspects of bacterial metabolism and growth, including the repression of DNA replication and rRNA synthesis and the concurrent enhancement of amino acid biosynthesis and uptake (1). Recent investigations have revealed that (pp)pGpp fulfills a wide spectrum of functions in bacterial adaptation to the limitations of carbon sources and fatty acids, antibiotic tolerance, biofilm formation, cell wall synthesis, encapsulation, heat shock, and motility (5–7). While the molecular interactions between (pp)pGpp and their targets have been extensively characterized, it is largely unknown how the stringent response rapidly inhibits bacterial growth while enhancing survival at the metabolomic level.

Streptococcus pneumoniae (the pneumococcus) is a commensal of the human nasopharynx and an opportunistic pathogen that causes various infections, including pneumonia, bacteremia, meningitis, and otitis media (8, 9). The rate of pneumococcal colonization in young children (the population most vulnerable to pneumococcal disease) can be as high as 100% (10, 11). It is poorly understood how S. pneumoniae colonizes the mucosal surface of the upper airway with scarce nutrients under host-imposed nutritional immunity (12, 13). Previous studies have shown that S. pneumoniae is auxotrophic for 8 amino acids (arginine, cysteine, histidine, glycine, glutamine, isoleucine, leucine, and valine) under *in vitro* conditions (14, 15). While a large number of putative amino acid transporters and synthesis pathways are predicted in the pneumococcal genome (16), details of amino acid uptake and biosynthesis in the bacterium are mostly unknown. The GlnPQ, *metQNP*, and arginine-ornithine antiporter loci are the three validated transporters for the uptake of glutamine (17), methionine (18), and arginine (19, 20), respectively. S. pneumoniae possesses a single RSH enzyme, Rel_spn_, which produces ppGpp and pppGpp after treatment with mupirocin, an inducer of the stringent response (15). In agreement with the role of the stringent response in bacterial survival, Rel_spn_ is dispensable for bacterial growth in complex medium but essential in chemically defined medium (CDM) (15).

Methionine plays many important roles in protein synthesis and the generation of *S*-adenosyl methionine (SAM) for the methylation of various macromolecules (21, 22). However, methionine is one of the rarest amino acids in physiological fluids (e.g., 4 μg/mL in human serum) (23). Our recent work has shown that S. pneumoniae employs the MetR transcriptional regulator to sense the level of the intracellular methionine pool (24). In a methionine-rich environment, S. pneumoniae fully depends on the MetQNP high-affinity methionine uptake system and an uncharacterized low-affinity transporter system(s) to acquire “free” methionine. In the absence of extracellular methionine, the bacterium synthesizes methionine with cysteine and folic acid by the MetR-mediated transcriptional activation of the methionine synthesis system, which includes MetB (cystathionine γ-synthase), MetC (aminotransferase), MetE (5-methyltetrahydropteroyltriglutamate-homocysteine *S*-methyltransferase), and MetF (5,10-methylenetetrahydrofolate reductase) (24, 25). The *metE* and *metR* deletion mutants are fully auxotrophic for methionine (24).

Previous studies have revealed the massive overhaul of the pneumococcal transcriptome under methionine starvation conditions (24, 25). The differentially expressed genes include not only those associated with methionine biosynthesis/utilization but also many others that do not have an apparent relationship with methionine metabolism. Transcriptomic analyses have thus indicated a fundamental reprogramming of pneumococcal metabolism under methionine starvation. In this paper, we report our systematic profiling of the pneumococcal metabolome under methionine-sufficient and methionine starvation conditions and, to the best of our knowledge, provide the first bacterial metabolome under amino acid starvation. These data have revealed many previously unrecognized features of bacterial metabolism in response to methionine starvation. In particular, the significant accumulation of glutamine and lactate appears to form a “prosurvival” metabolic state with a lower intracellular pH, which inhibits bacterial growth for prolonged survival.

## RESULTS

### Methionine starvation enhances pneumococcal survival.

**FIG 1:**
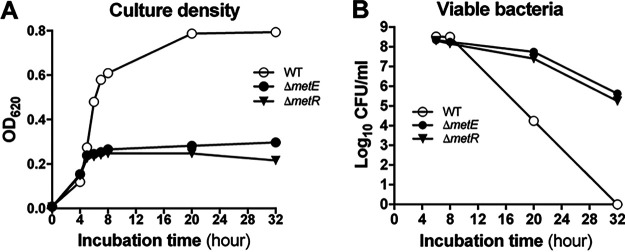
Induction of pneumococcal survival by methionine starvation. (A) Growth of S. pneumoniae D39 and its methionine synthesis-deficient Δ*metE* and Δ*metR* mutants under methionine starvation. Pneumococcal growth in chemically defined medium (CDM) with 1 μg/mL methionine was assessed by the optical density at 620 nm (OD_620_) at various time points after inoculation. (B) Survival of the D39, Δ*metE*, and Δ*metR* strains under methionine starvation. Viable pneumococci in the cultures from panel A were quantified as CFU, which were determined by spreading serially diluted cultures onto blood agar plates starting at 6 h when the mutants reached a maximal density.

Our previous work shows that S. pneumoniae activates MetE-dependent methionine synthesis by MetR (a LysR family transcriptional regulator) when the amino acid supply is limited (e.g., 1 μg/mL methionine) (24). In CDM with 1 μg/mL methionine, wild-type (WT) strain D39 showed robust growth with a maximal optical density at 620 nm (OD_620_) of 0.79, but the mutants lacking the *metR* (Δ*metR*) or *metE* (Δ*metE*) gene displayed relatively poor growth (Fig. 1A). This result showed that this methionine concentration represents severe methionine starvation conditions for the Δ*metR* and Δ*metE* mutants. *metE* encodes methionine synthase, which transfers the methyl group from 5-methyl tetrahydrofolate (a form of folic acid) to l-homocysteine to form l-methionine (26). While S. pneumoniae cells are prone to stationary-phase lysis or autolysis (reduction in the culture density) in complex medium (e.g., Todd-Hewitt broth supplemented with yeast extract [THY broth]) (27, 28), for an unknown reason(s), D39 and its *metR* and *metE* mutants did not display obvious autolysis in CDM at stationary phase. This allowed us to compare the survival rates of different strains under methionine starvation.

To determine the survival capacity, we enumerated the CFU of D39 and its Δ*metE* and Δ*metR* mutants with methionine synthesis deficiency at various times of incubation. In keeping with their comparable OD_620_ values, the cultures of the WT (3.3 × 10^8^ CFU/mL), Δ*metE* (2.2 × 10^8^ CFU/mL), and Δ*metR* (2.1 × 10^8^ CFU/mL) strains showed high levels of viable bacteria at 6 h (Fig. 1B). However, the D39 CFU value was dramatically diminished by 4.3-log_10_-fold from 6 to 20 h, indicating that the bacteria died without cellular lysis. In sharp contrast, a much slower loss of viability was observed for the Δ*metE* (by 2.9-fold) and Δ*metR* (by 7.3-fold) mutants from 6 to 20 h. The CFU values of the Δ*metE* and Δ*metR* mutants were 3.5-log_10_-fold and 3.2-log_10_-fold higher than that of D39 at 20 h, respectively. Consistently, no viable D39 bacteria were detected at 32 h, but there were substantial levels of viable bacteria in the cultures of the Δ*metE* (4.1 × 10^5^ CFU/mL) and Δ*metR* (1.7 × 10^5^ CFU/mL) mutants at that time point. These data revealed that severe methionine starvation restrains the growth but enhances the survival of pneumococci. Pneumococcal autolysis does not appear to affect the methionine starvation-induced pneumococcal survival, since deleting *lytA* in Δ*metE*, encoding the major autolysin in *S. pneumoniae*, did not significantly alter growth kinetics (Fig. S1A) or survival (Fig. S1B) of the bacterium.

10.1128/msphere.00625-22.1 FIG S1 Impact of *lytA* deletion on pneumococcal growth and survival. Growth (A) and survival (B) of S. pneumoniae D39 Δ*metE* and its Δ*lytA* mutant (Δ*metE* Δ*lytA*) are shown. The cells were cultivated in chemically defined medium (CDM) with 200 μg/mL methionine. Pneumococcal growth was determined as described in the legend to Fig. 1A. Viable pneumococci of the cultures were quantified by CFU as in Fig. 1B. Download FIG S1, TIF file, 1.3 MB. Copyright © 2023 Zhang et al. 2023 Zhang et al. https://creativecommons.org/licenses/by/4.0/ This content is distributed under the terms of the Creative Commons Attribution 4.0 International license.

### Methionine starvation induces a massive overhaul of the pneumococcal metabolome.

**FIG 2:**
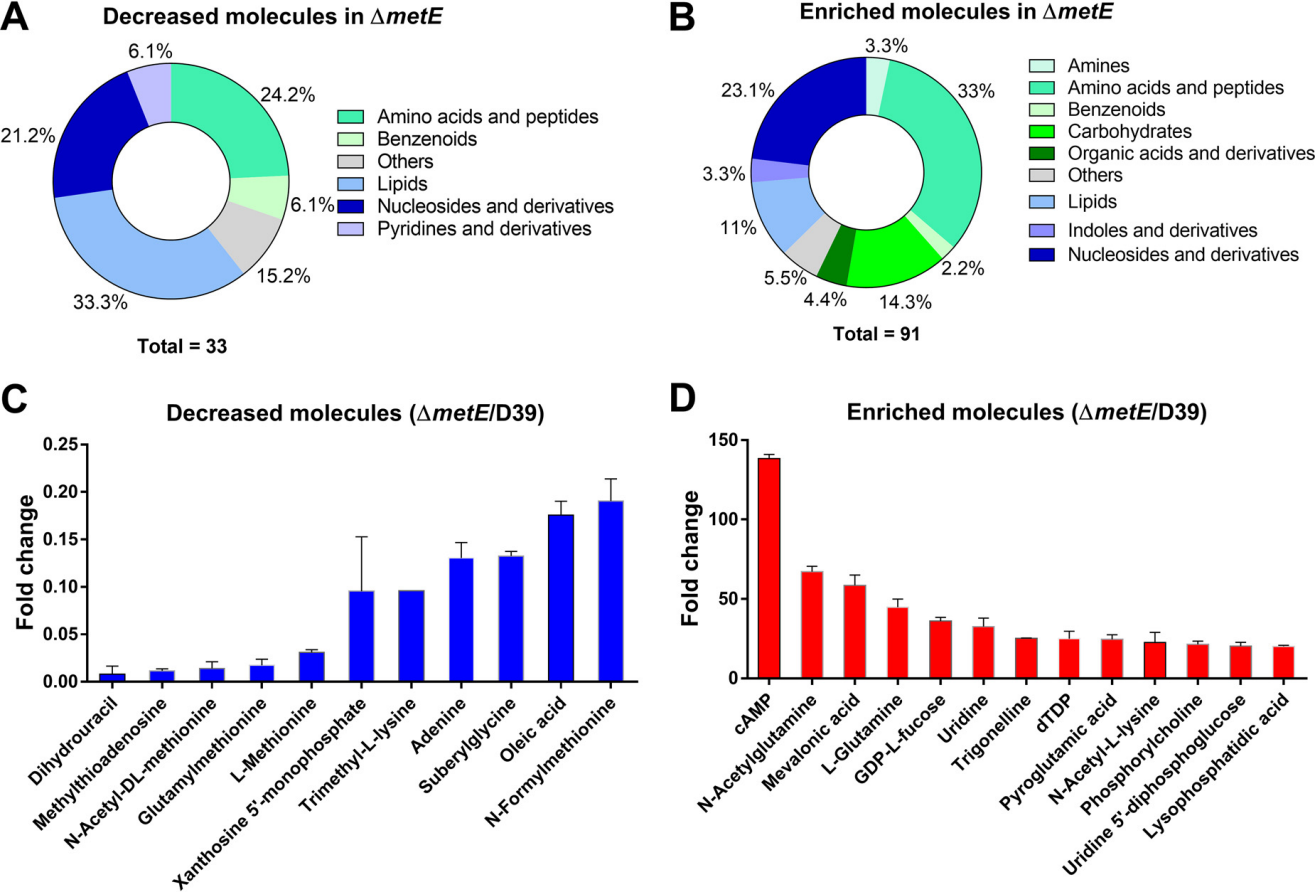
Metabolomic changes in S. pneumoniae D39 under methionine starvation. (A and B) Classification of decreased (A) and enriched (B) metabolites in the metabolome of the Δ*metE* mutant compared with the parental strain D39. Bacteria were cultivated in DCM with 1 μg/mL methionine for 6 h. (C and D) Most significantly regulated metabolites in the metabolome of the Δ*metE* mutant. Metabolites that were decreased by at least 5-fold (C) or enriched by at least 20-fold (D). The metabolomes of D39 and the Δ*metE* mutant were determined after cultivation for 6 h in CDM supplemented with 1 μg/mL methionine. The complete data are available in Table S1 in the supplemental material.

To understand how methionine starvation induces pneumococcal survival, we compared the metabolomes of D39 and the Δ*metE* mutant after the bacteria were cultivated for 6 h in CDM with 1 μg/mL methionine. This analysis revealed a striking overhaul of the pneumococcal metabolome under methionine starvation. A total of 305 metabolites were identified by metabolomic profiling (see Table S1 in the supplemental material). With a cutoff combination of a 0.5-fold-higher or -lower value than that of the WT and a *P* value (by Student’s *t* test) of <0.05, 124 molecules were identified as significantly decreased (Fig. 2A) or increased (Fig. 2B) in the Δ*metE* mutant. As expected, many metabolites related to methionine metabolism were among the ones most dramatically decreased in the Δ*metE* mutant (Fig. 2C), which included l-methionine and methionine derivatives (e.g., 5′-methylthioadenosine, *N*-acetyl-dl-methionine, glutamylmethionine, and *N*-formylmethionine). A number of fatty acids were substantially reduced, such as oleic acid, linoleic acid, and palmitoleic acid. In the context of the dramatic increase in mevalonate (Fig. 2D) (see below), this result indicated cellular disorders in lipid metabolism and membrane stability under methionine starvation.

10.1128/msphere.00625-22.5 TABLE S1 Pneumococcal metabolites detected by LC-MS/MS in D39 and the *metE* mutant at 1 μg/mL methionine. Biological duplicates were included in the analysis. Metabolite abundance is displayed for relative quantitation after normalization by protein concentration. Fold changes were calculated using the average intensities of duplicates. Download Table S1, XLSX file, 0.05 MB. Copyright © 2023 Zhang et al. 2023 Zhang et al. https://creativecommons.org/licenses/by/4.0/ This content is distributed under the terms of the Creative Commons Attribution 4.0 International license.

Cyclic AMP (cAMP) (by 137-fold), *N*-acetylglutamine (by 66-fold), mevalonate (by 58-fold), l-glutamine (by 44-fold), and GDP-l-fucose (by 35-folds) are the top 5 enriched molecules in the Δ*metE* mutant (Fig. 2D). The abundance of cAMP might have resulted from the methionine starvation-associated stress response (cAMP) since it has been linked to the regulation of cellular (p)ppGpp (29), an essential second messenger in limitations of amino acids and other nutrients (30). The overwhelming accumulation of mevalonate suggested metabolic disorders in the mevalonate pathway, which is highly conserved in all forms of life and generates isoprenoid compounds for many cellular functions in bacteria, such as cell wall synthesis, capsule synthesis, membrane integrity, electron transport, and protein modification (31). The mevalonate pathway in S. pneumoniae is essential for *in vitro* growth (32) and cell division (33). Consistently, a number of cell wall synthesis precursors also accumulated in the Δ*metE* mutant, including UDP-glucose, phosphorylcholine, and UDP-*N*-acetylglucosamine. l-Glutamine and l-glutamic acid (by 4.1-fold) were the two amino acids with the most dramatic increases in the Δ*metE* mutant (Table S1). This result is consistent with the substantial increases in numerous derivatives of l-glutamine (*N*-acetylglutamine) and l-glutamic acid (alanylglutamic acid, glutaminylglutamic acid, leucylglutamic acid, pyroglutamic acid, *N*-acetylaspartylglutamic acid, *N*-acetylglutamic acid, and valylglutamic acid).

Because the Δ*metR* and Δ*metE* mutants showed similar patterns of growth and survival under methionine starvation, we characterized the impact of methionine starvation on the pneumococcal metabolome in the Δ*metR* mutant. A remarkably similar pattern of metabolomic changes was observed for the Δ*metR* mutant (Table S2 and Fig. S2A and B). These included dramatic reductions in l-methionine and its derivatives (Fig. S2C) and abundant increases in mevalonate (by 42-fold), cAMP (by 38-fold), and GDP-l-fucose (by 29-fold) (Fig. S2D). l-Glutamine (by 31-fold) and l-glutamic acid (by 4.2-fold) were also the two amino acids with the highest abundances in the Δ*metR* mutant. The mutant also displayed more abundant *N*-acetylglutamine (by 40-fold) and the same 7 l-glutamic acid derivatives enriched in the Δ*metE* mutant. The metabolomic data have revealed that methionine starvation induces a dramatic and robust reprogramming of the pneumococcal metabolome. Based on the massive increase in intracellular glutamine under methionine starvation conditions, our subsequent investigation focused on the functional impact of glutamine on bacterial adaptation to methionine limitation.

10.1128/msphere.00625-22.2 FIG S2 Metabolomic changes in S. pneumoniae Δ*metR* mutant under methionine starvation. (A and B) Classification of decreased (A) and enriched (B) metabolites in the metabolome of the Δ*metR* mutant compared with the parental strain D39. Bacteria were cultivated in DCM with 1 μg/ml methionine for 6 h. (C and D) Most significantly regulated metabolites in the metabolome of the Δ*metR* mutant. Metabolites that were decreased by at least 5-fold (C) or enriched by at least 20-fold (D) are indicated. The metabolomes of D39 and the Δ*metR* mutant were determined after cultivation for 6 h in CDM supplemented with 1 μg/mL methionine. The complete data are available in Table S2 in the supplemental material. Download FIG S1, TIF file, 0.6 MB. Copyright © 2023 Zhang et al. 2023 Zhang et al. https://creativecommons.org/licenses/by/4.0/ This content is distributed under the terms of the Creative Commons Attribution 4.0 International license.

10.1128/msphere.00625-22.6 TABLE S2 Pneumococcal metabolites detected by LC-MS/MS in D39 and the *metR* mutant at 1 μg/mL methionine. Biological duplicates were included in the analysis. Metabolite abundance is displayed for relative quantitation after normalization by protein concentration. Fold changes were calculated using the average intensities of duplicates. Download Table S2, XLSX file, 0.05 MB. Copyright © 2023 Zhang et al. 2023 Zhang et al. https://creativecommons.org/licenses/by/4.0/ This content is distributed under the terms of the Creative Commons Attribution 4.0 International license.

### Methionine starvation induces the abundant accumulation of intracellular glutamine.

**FIG 3:**
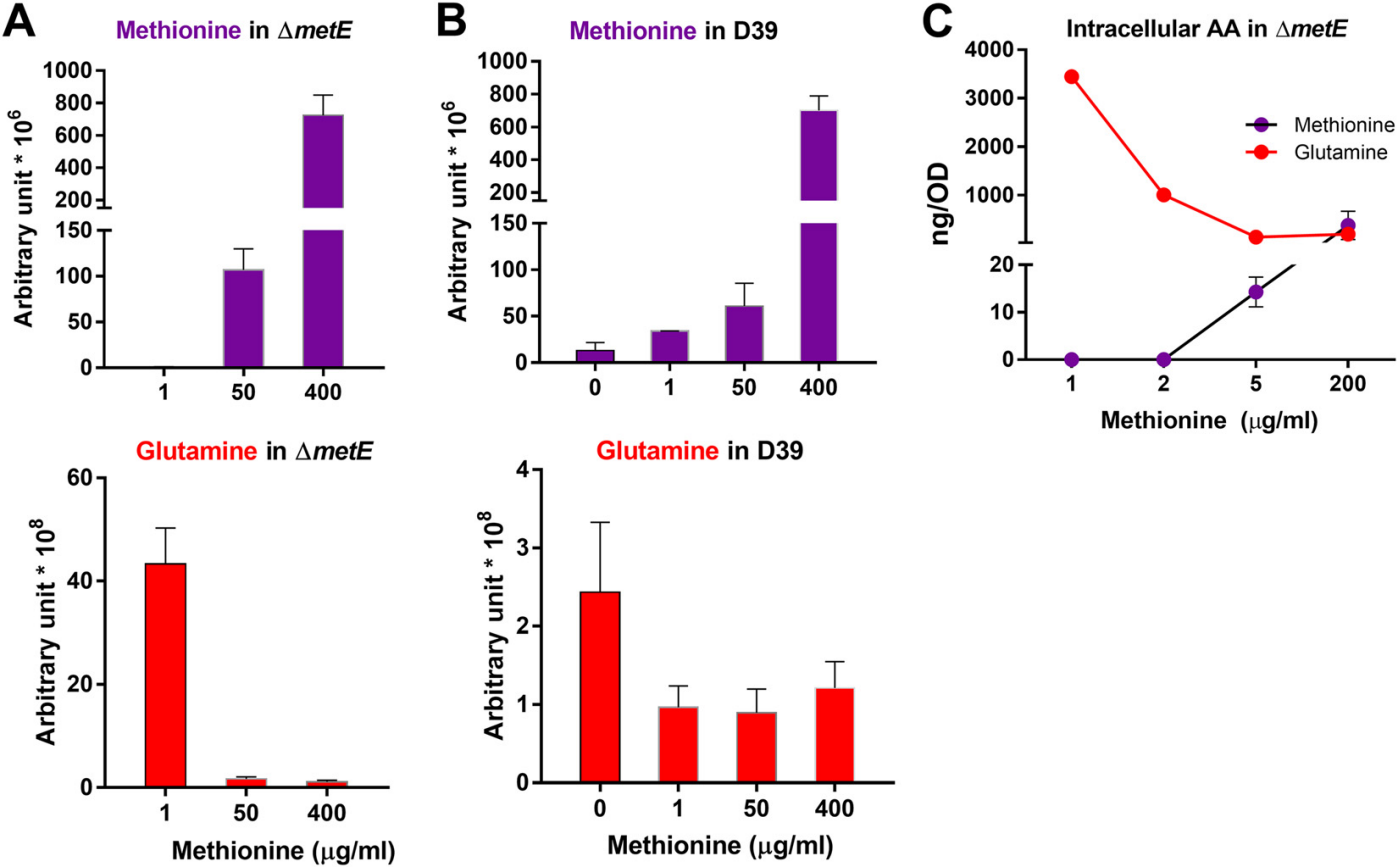
Methionine concentration-dependent accumulation of intracellular glutamine. (A) Inverse relationship between the intracellular levels of methionine and glutamine in the Δ*metE* mutant. The Δ*metE* metabolome was determined after cultivation for 6 h in CDM supplemented with 1, 50, or 400 μg/mL methionine. The levels of intracellular methionine and glutamine determined by metabolic analysis are shown at the top and bottom, respectively. The complete data are available in Table S3 in the supplemental material. (B) Inverse relationship between the intracellular levels of methionine and glutamine in D39. The D39 metabolome was characterized with CDM cultures supplemented with 0, 1, 50, or 400 μg/mL methionine and is presented as described above for panel A. The complete data are available in Table S4. (C) Specific quantification of intracellular methionine and glutamine in the Δ*metE* mutant. Bacteria were cultured in CDM with 1, 2, 5, or 200 μg/mL methionine for 6 h and collected for the targeted quantification of intracellular methionine and glutamine by LC-tandem MS (MS/MS). The results are presented as nanograms per OD_620_ unit of the original culture. AA, amino acid.

To verify the relationship between methionine starvation and the massive accumulation of intracellular glutamine, we further determined the metabolome of the Δ*metE* mutant cultivated in CDM with 1, 50, and 400 μg/mL methionine. The combination of liquid chromatography (LC) and mass spectrophotometry (MS) revealed an inverse relationship between the methionine concentration in CDM and the abundance of intracellular glutamine in the Δ*metE* mutant (Table S3). Comparative analysis showed that the methionine synthesis-deficient mutant grown with 1 μg/mL methionine showed the lowest level of intracellular methionine (Fig. 3A, top) and the highest level of glutamine (Fig. 3A, bottom). A further increase in the methionine concentration to 50 μg/mL led to a 106-fold increase in intracellular methionine and a 25-fold reduction in intracellular glutamine. When methionine was supplemented at 400 μg/mL, the intracellular methionine level was further increased by an additional 6-fold, but the level of intracellular glutamine remained unchanged. In keeping with this result, the Δ*metE* mutant showed a growth pattern comparable to that of D39 in CDM with 50 and 400 μg/mL methionine (24), indicating that environmental methionine at 50 μg/mL is sufficient to support the normal growth of S. pneumoniae in the absence of methionine synthesis. These changes in the metabolome of the Δ*metE* mutant with various concentrations of methionine in CDM confirmed that methionine starvation induces the accumulation of intracellular glutamine.

10.1128/msphere.00625-22.7 TABLE S3 Pneumococcal metabolites detected by LC-MS/MS in D39 Δ*metE* at 1, 50, and 400 μg/mL methionine. Bological duplicates were included in the analysis. Metabolite abundance is displayed for relative quantitation after normalization by protein concentration. Fold changes were calculated using the average intensities of duplicates. Download Table S3, XLSX file, 0.05 MB. Copyright © 2023 Zhang et al. 2023 Zhang et al. https://creativecommons.org/licenses/by/4.0/ This content is distributed under the terms of the Creative Commons Attribution 4.0 International license.

10.1128/msphere.00625-22.8 TABLE S4 Pneumococcal metabolites detected by LC-MS/MS in D39 at 0, 1, 50, and 400 μg/mL methionine. Biological duplicates were included in the analysis. Metabolite abundance is displayed for relative quantitation after normalization by protein concentration. Fold changes were calculated using the average intensities of duplicates. Download Table S4, XLSX file, 0.07 MB. Copyright © 2023 Zhang et al. 2023 Zhang et al. https://creativecommons.org/licenses/by/4.0/ This content is distributed under the terms of the Creative Commons Attribution 4.0 International license.

The methionine starvation-induced accumulation of intracellular glutamine was also observed for the metabolome of D39 cultivated in CDM with 0, 1, 50, and 400 μg/mL methionine (Table S4). The intracellular methionine concentration increased proportionally with additional supplementation with the amino acid in CDM, as exemplified by the 52-fold increase in intracellular methionine between the culture conditions of 0 and 400 μg/mL methionine (Fig. 3B, top). In contrast to the dramatic accumulation of glutamine in the Δ*metE* mutant with 1 μg/mL methionine compared with methionine-sufficient conditions (Fig. 3A), there was a similar level of intracellular glutamine in D39 cells cultivated with 1, 50, and 400 μg/mL methionine (Fig. 3B, bottom). The glutamine concentration only doubled between the culture conditions of 0 and 400 μg/mL methionine, which agrees with our previous observation that normal methionine synthesis alone in D39 is nearly sufficient for bacterial growth (24). This result showed that a mild methionine shortage induces a modest accumulation of intracellular glutamine.

We finally performed selective quantification of intracellular methionine and glutamine in the D39 and Δ*metE* strains cultivated in CDM with various concentrations of methionine. An inverse relationship between methionine supplementation and intracellular glutamine was observed. The culture with 1 μg/mL methionine showed the highest level of glutamine (3,446.5 ng/OD), without detectable intracellular methionine (Fig. 3C). When methionine was supplemented at 200 μg/mL, a standard concentration in CDM (34), intracellular methionine was increased to 377.4 ng/OD. In an opposite fashion, the glutamine level was reduced to 198.2 ng/OD, a 16.4-fold reduction compared with the level with 1 μg/mL methionine. This analysis showed that methionine starvation induces the accumulation of intracellular glutamine in a concentration-dependent manner.

### Intracellular glutamine accumulation enhances pneumococcal survival.

**FIG 4:**
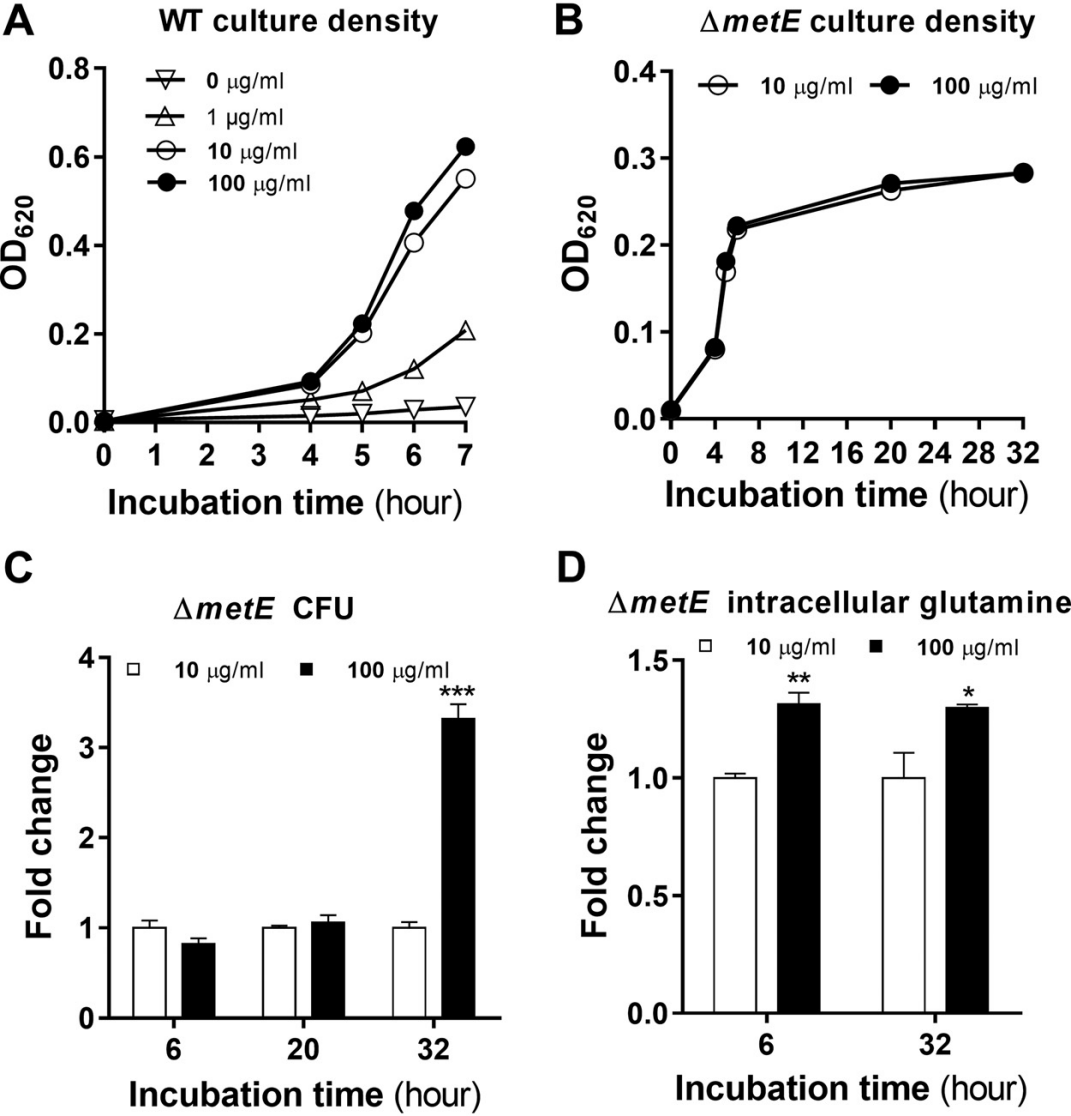
Enhancement of pneumococcal survival by glutamine under methionine starvation. (A) Impact of glutamine availability on pneumococcal growth with sufficient methionine. D39 cells were cultivated in CDM with 200 μg/mL methionine and various concentrations of glutamine. Bacterial growth is presented as optical density values (OD_620_) at various time points. (B) Growth characteristics of methionine-starved Δ*metE* cells. Methionine synthesis-deficient Δ*metE* cells were grown in CDM with 1 μg/mL methionine and 10 μg/mL or 100 μg/mL glutamine. Bacterial growth is presented as optical density values (OD_620_) at various time points. (C) Glutamine-enhanced survival of methionine-starved Δ*metE* cells. The viable bacteria in the Δ*metE* culture from panel B were enumerated as CFU at various times, as described in the legend of Fig. 1B. (D) Effect of extracellular glutamine on the intracellular glutamine level in methionine-starved Δ*metE* cells. Intracellular glutamine levels in the Δ*metE* culture from panel B were assessed by metabolomic procedures as described in Materials and Methods and are presented as fold changes between 10 μg/mL and 100 μg/mL glutamine.

On the basis of the significant increase in bacterial survival under methionine starvation during stationary phase, the massive accumulation of intracellular glutamine suggested a significant contribution of glutamine to pneumococcal adaptation. Glutamine is one of the 8 essential amino acids that are unable to be synthesized by S. pneumoniae D39 (14, 15). We first evaluated pneumococcal growth in CDM with various concentrations of glutamine and 200 μg/mL methionine (a concentration in CDM). D39 showed a growth pattern with a strict dependence on the glutamine concentration (Fig. 4A, 0 μg/mL). While no growth was observed in the absence of glutamine (0 μg/mL), 10 μg/mL glutamine supported normal growth compared with 100 μg/mL glutamine, a specified concentration in CDM (34); a further decrease in the glutamine concentration (1 μg/mL) substantially impaired pneumococcal growth. We thus tested the impact of glutamine on pneumococcal adaptation by comparing pneumococcal survival at 10 μg/mL and 100 μg/mL glutamine under methionine starvation (1 μg/mL). The Δ*metE* cultures with both glutamine concentrations showed comparable growth with 1 μg/mL methionine, as reflected by the virtually identical patterns of the optical densities of the two cultures at various time points (Fig. 4B). However, the higher concentration of glutamine conferred survival far superior to that of the low-concentration counterpart at the late stationary phase (Fig. 4C). The CFU count of the culture with 100 μg/mL glutamine was 3.3-fold higher than that of its counterpart with 10 μg/mL glutamine at 32 h. In agreement with the survival results, pneumococcal cells in CDM with 100 μg/mL glutamine possessed higher levels of intracellular glutamine under methionine starvation conditions (Fig. 4D). These data showed that the accumulation of intracellular glutamine is critical for pneumococcal survival under methionine starvation.

### The GlnPQ ABC transporter is involved in intracellular glutamine accumulation.

**FIG 5:**
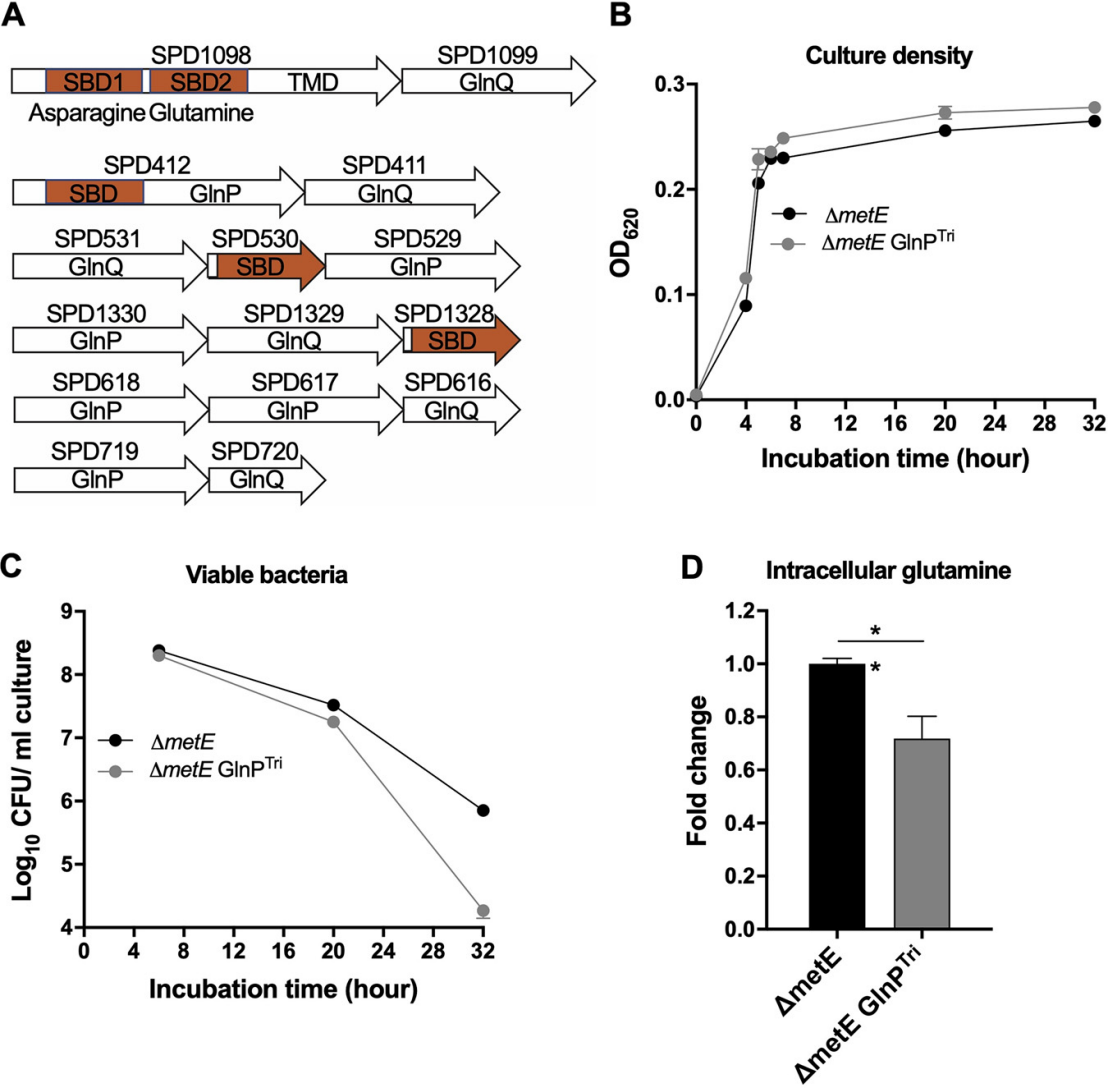
Contribution of the SPD1098-1099 locus to glutamine accumulation under methionine starvation. (A) Diagram of predicted glutamine transporters in S. pneumoniae. SBD, TMD, GlnP, and GlnQ represent the substrate binding domain, transmembrane domain, permease, and ATPase, respectively. (B and C) Growth (B) and survival (C) of the Δ*metE* and Δ*metE* GlnP^Tri^ mutants in CDM with 1 μg/ml methionine. (D) Effect of GlnP point mutations on the intracellular glutamine level of the methionine-starved Δ*metE* mutant. All bacteria were grown in CDM with 1 μg/mL methionine for 6 h. Intracellular glutamine levels were measured by LC-MS/MS as described in Materials and Methods.

The requirement of glutamine supplementation in CDM for pneumococcal growth and survival indicated that intracellular accumulation of glutamine occurs via amino acid uptake. A previous study has predicted 6 glutamine transporter loci in the genome of S. pneumoniae (35) (Fig. 5A), but the SPD1098-1099 locus is shown to recognize glutamine (17). Except for the SPD616-618 and SPD719-720 loci, the other four systems contain a substrate binding domain (SBD), a permease (GlnP) or a transmembrane domain (TMD), and an ATPase (GlnQ). In SPD1098-1099 and SPD411-412, the SBDs are fused to a TMD to form SPD1098 and SPD411, respectively. Interestingly, SPD1098 contains two SBDs, SBD1 for asparagine binding and SBD2 for glutamine binding (17).

To ascertain the glutamine transporter(s) responsible for intracellular glutamine accumulation in methionine-starved *S. pneumoniae*, we tested *in vitro* glutamine binding of the known glutamine-binding SBD2 in SPD1098 and 4 putative SBDs using isothermal titration calorimetry (ITC). Consistent with the previous study (1), SBD2 and SBD1 of SPD1098 bound to glutamine and asparagine, respectively (Table 1). By contrast, none of the other putative SBDs showed obvious glutamine binding. We further characterized the functional contribution of SPD1098 to glutamine accumulation by targeting the three amino acids in SBD2 that are known for glutamine binding (F277, S335, and S384). While the triple mutations (F277Y, S335T, and S384A) in the Δ*metE* background (Δ*metE* GlnP^Tri^) did not impair bacterial growth (Fig. 5B), the CFU count of the mutant was decreased by approximately 40-fold at 32 h postinoculation compared with the parental strain (Fig. 5C). Consistent with the impaired bacterial survival, the intracellular glutamine concentration was reduced by 28% (Fig. 5D). These results have allowed us to conclude that the GlnPQ is mainly responsible for glutamine accumulation under methionine starvation.

**TABLE 1:** Glutamine or asparagine binding affinities of putative glutamine transporters^*a*^

Gene ID	Domain	Protein concn (μM)	Ligand	Amino acid concn (μM)	*K_D_* (μM)
SPD1098	SBD1	41	Asn	500	1.09
	SBD2	35	Gln	500	0.89
	SBD2	35	Asn	500	No fitting
SPD0412	SBD	41	Gln	500	No fitting
	SBD	82	Gln	1,500	No fitting
SPD0530	SBD	32	Gln	500	No fitting
	SBD	60	Gln	1,000	No fitting
SPD1328	SBD	71	Gln	1,000	No fitting
	SBD	71	Gln	1,500	No fitting

a Gln, glutamine; Asn, asparagine; *K_D_*, dissociation constant.

### Glutamine enhances pneumococcal survival by balancing the cellular pH.

**FIG 6:**
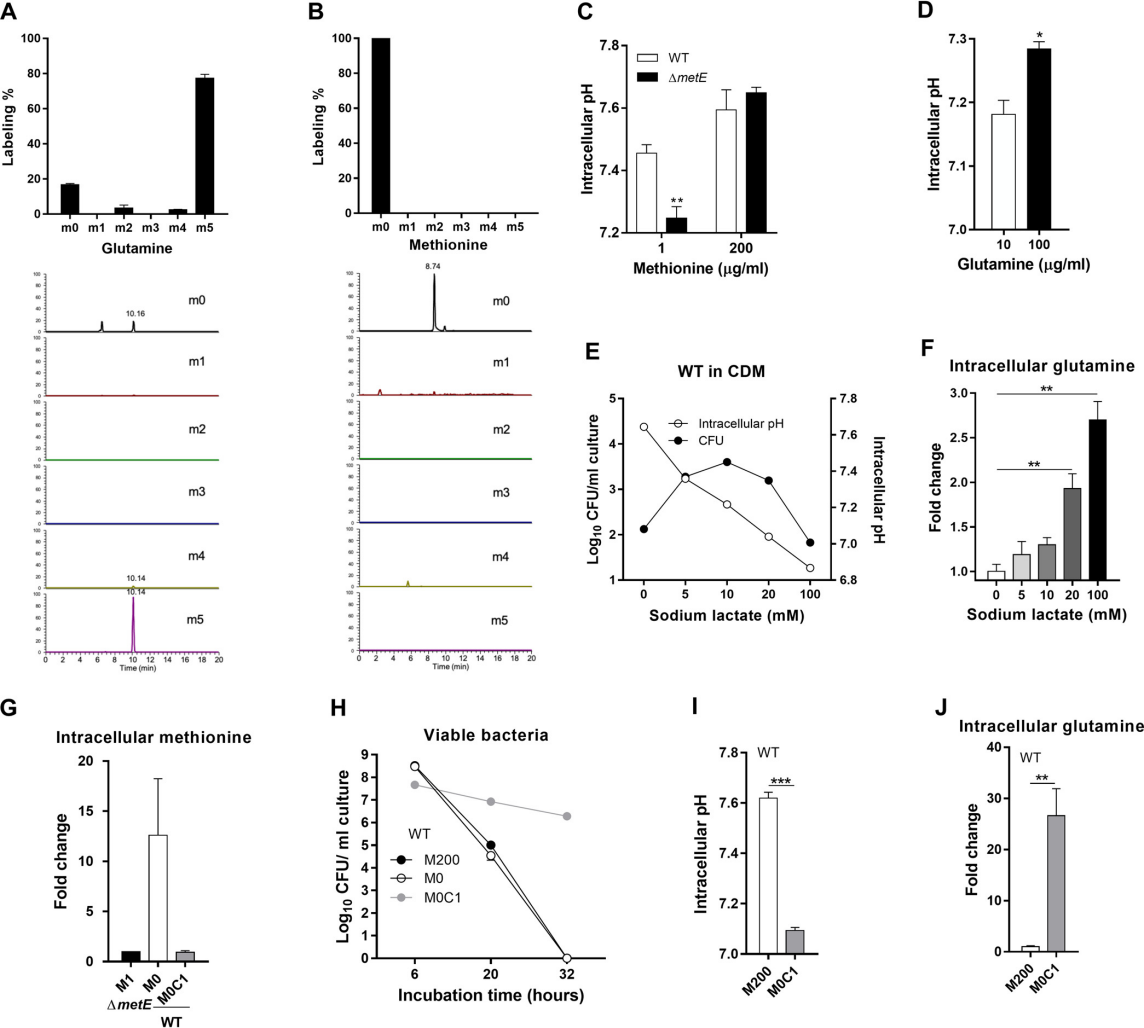
Modulation of cellular pH by glutamine accumulation. (A) Importance of amino acid uptake in the intracellular glutamine pool. D39 cells were grown in CDM with 200 μg/mL methionine and 100 μg/mL [^13^C_5_]glutamine for 6 h before the measurement of the relative abundance of intracellular glutamine without (m0) or with 1 to 5 (m1 to m5) ^13^C labels. The data are presented as percentages of each glutamine species of the total amino acids (top) or relative abundances in chromatography columns (bottom). The mass-to-charge ratios of glutamine are *m/z* 147.0769, 148.0802, 149.0836, 150.0869, 151.0903, and 152.0936 for m0 to m5, respectively. (B) The lack of ^13^C labeling in cellular methionine. The procedures were the same as the ones described above for panel A except for the ^13^C-labeled detection of methionine. The mass-to-charge ratios of methionine are *m/z* 150.0589, 151.0622, 152.0656, 153.0689, 154.0723, and 155.0756 for m0 to m5, respectively. (C) Cellular pH of D39 (WT) and the Δ*metE* mutant under methionine starvation. Pneumococci were cultured in CDM with 1 μg/mL or 200 μg/mL methionine for 20 h before cellular pH determination. (D) Impact of extracellular glutamine on the cellular pH of methionine-starved Δ*metE* cells. D39 (WT) and Δ*metE* cells were cultured in CDM with 10 μg/mL or 100 μg/mL glutamine under methionine starvation (1 μg/mL) for 20 h before cellular pH determination. (E) Relationship between the lactate level and pneumococcal survival/cellular pH. D39 cells were grown in CDM for 6 h before supplementation with sodium lactate at various concentrations. Cellular pH measurement and CFU counting were carried out at 20 and 32 h, respectively. (F) Impact of the lactate level on intracellular glutamine. The D39 cultures from panel E were processed to determine intracellular glutamine levels by metabolomic procedures. Differences between the cultures with and those without sodium lactate supplementation in intracellular glutamine are expressed as fold changes, in which the amino acid level in the culture without sodium lactate is set to a value of 1. (G) Effect of extracellular methionine with or without cysteine starvation on intracellular methionine level of D39 and Δ*metE*. Pneumococci were cultivated in DCM with 1 μg/ml methionine for Δ*metE* (M1) and 0 μg/ml methionine for D39 cells (M0). Additionally, methionine and cysteine costarved D39 cells were cultured in DCM with 0 μg/ml methionine and 1 μg/ml cysteine (M0C1). Intracellular methionine levels were measured after being cultivated for 20 hr. H. Enhanced survival of methionine-starved D39 under cysteine deficiency. The D39 cells were grown in CDM with 200 μg/ml and 0 μg/ml methionine (M200 and M0 respectively). Methionine and cysteine co-starved D39 cells were cultured in DCM with 0 μg/ml methionine and 1 μg/ml cysteine (M0C1). The viable bacteria in the D39 culture were counted as CFU at various time points. (I) Cellular pH of D39 under methionine and cysteine costarvation. Pneumococci were cultured in CDM with 200 μg/ml methionine (M200) and 1 μg/ml cysteine with no methionine (M0C1), respectively. The intracellular pH values were measured after cultivation for 20 h. (J) Impact of methionine and cysteine costarvation on intracellular glutamine level of D39. The cells were cultured as described for panel I. Intracellular glutamine was measured by LC-MS/MS as described in Materials and Methods, and the level of D39 in DCM with 200 μg/ml methionine is set as 1.

To determine how intracellular glutamine accumulation under methionine starvation enhances pneumococcal survival, we first tested the possibility that glutamine compensates for methionine deficiency by biochemical conversion to methionine using an isotope-tracing approach. The methionine synthesis-deficient pneumococci (Δ*metR*) were cultivated in CDM supplemented with 1 μg/mL methionine and 100 μg/mL [^13^C_5_]glutamine (in place of glutamine), in which ^13^C occupied all 5 carbon atoms of glutamine. At 6 h, the vast majority of intracellular glutamine molecules (77.3%) from the [^13^C_5_]glutamine CDM culture were found to carry ^13^C at all 5 carbon positions (m5) (Fig. 6A), indicative of glutamine uptake. The ^13^C-free glutamine (m0 species) (16.7%) might represent the preexisting amino acid pool before labeling and, possibly, the contribution of the putative glutamine synthetase GlnRA, as indicated previously (36, 37). No ^13^C-labeled methionine was detected from the methionine-starved pneumococci (Fig. 6B). This result demonstrated that S. pneumoniae is unable to synthesize methionine from glutamine and depends mainly on uptake to replenish intracellular glutamine.

Lu et al. reported previously that l-glutamine promotes the acid resistance of E. coli by neutralizing intracellular protons via releasing ammonium (38). We thus tested if glutamine impacts the cellular pH of S. pneumoniae under methionine starvation using a pH-sensitive green fluorescent protein (GFP) (pH-GFP) (39). The cellular pHs of the D39 (pH 7.5) and Δ*metE* (pH 7.6) strains were similar when cultivated in methionine-rich CDM (200 μg/mL) (Fig. 6C). However, the Δ*metE* mutant displayed a significantly lower pH (pH ~7.2) than that of the WT (pH 7.5) under methionine starvation (1 μg/mL). The methionine concentration-dependent reduction in the cellular pH could be significantly dampened by glutamine supplementation in CDM (Fig. 6D). The cellular pH of the Δ*metE* mutant grown with 100 μg/mL glutamine was substantially higher than that of its counterpart with 10 μg/mL glutamine under methionine starvation.

Methionine starvation-elicited intracellular acidification was also consistent with the significantly higher levels of intracellular lactate in the metabolomes of the Δ*metE* and Δ*metR* mutants with 1 μg/mL methionine. The levels of lactate in the Δ*metE* and Δ*metR* mutants were 3.0-fold higher than that in the WT under methionine starvation (Tables S1 and S2). Consistently, multiple intermediate metabolites of glycolysis were also increased in both the Δ*metE* and Δ*metR* mutants, such as glucose-6-phosphate, fructose 1,6-bisphosphate, and glyceraldehyde 3-phosphate. Since lactate is known to be a key factor in reducing the cellular pH (40), we assessed the impact of lactate on the pneumococcal cellular pH and survival by adding 5 to 100 mM sodium lactate to D39 cultures in standard CDM at 6 h. Intracellular lactate level was successfully increased by addition of sodium lactate in CEM (Fig. S3A). With supplementation with sodium lactate at final concentrations of up to 100 mM, the intracellular lactate rose from 185 to 1,939 ng/OD. The pyruvate level was also elevated along with lactate addition (Fig. S3B), suggesting the conversion of lactate to intracellular pyruvate. The cellular pH was proportionally reduced by increasing the lactate concentration in CDM at 20 h, ranging from 7.6 (0 mM) to 6.9 (100 mM) (Fig. 6E). Maximal bacterial survival (4,000 CFU) at 32 h was observed at pH 7.2 when lactate was supplemented at a final concentration of 10 mM. Bacterial survival was significantly impaired when the cellular pH was shifted either above or below pH 7.2 by lactate supplementation, as exemplified by 134 CFU and 67 CFU for the pneumococci with intracellular pHs of 7.6 and 6.9, respectively. This result strongly suggested that pH 7.2 is the optimal cellular pH for pneumococcal survival under methionine starvation. Consistent with intracellular acidification and glutamine accumulation under methionine starvation, the addition of lactate to the D39 culture not only reduced the cellular pH (Fig. 6E) but also simultaneously increased the intracellular glutamine concentration in a dose-dependent manner (Fig. 6F).

10.1128/msphere.00625-22.3 FIG S3 Enhancement of intracellular lactate and pyruvate of pneumococcal D39 by addition of extracellular sodium lactate. Shown is the impact of extracellular lactate on intracellular levels of lactate (A) and pyruvate (B) of pneumococcal D39. Pneumococcal cells were cultured in CDM with the addition of 0, 10, or 100 mM sodium lactate as described in the legend to Fig. 6E and F. Levels of lactate and pyruvate were measured by LC-MS/MS at 20 h. Download FIG S2, TIF file, 0.6 MB. Copyright © 2023 Zhang et al. 2023 Zhang et al. https://creativecommons.org/licenses/by/4.0/ This content is distributed under the terms of the Creative Commons Attribution 4.0 International license.

To exclude an unexpected effect of the gene deletion of *metE*, the response to methionine starvation was further investigated in the WT. Reducing the level of methionine itself in medium failed to create starvation conditions in the WT. Even in the absence of cultural methionine (M0), the intracellular abundance of methionine in the WT was still 12-fold higher than that in the Δ*metE* mutant (Fig. 6G). Meanwhile, no observable survival enhancement was detected by removing the methionine supply (Fig. 6H, M0). Methionine biosynthesized from cysteine appeared to be sufficient for bacteria to avoid starvation. Therefore, additional cysteine restriction with 1 μg/mL in DCM was applied to achieve methionine-starved conditions, in which the intracellular methionine level of the WT was similar to that of the Δ*metE* mutant at 20 h postinoculation (Fig. 6G, M0C1). In this case, a significant survival improvement was observed (Fig. 6H, M0C1), confirming the enhanced survival of methionine-starved pneumococci. Consistently, an acidic intracellular environment (pH ~7.2) (Fig. 6I) and glutamine accumulation (Fig. 6J) were induced with colimitation methionine and cysteine. Taken together, these data indicated that massive glutamine uptake under methionine starvation enhances pneumococcal survival by maintaining a prosurvival cellular pH.

### Glutamine deaminases contribute to pneumococcal survival under methionine starvation.

**FIG 7:**
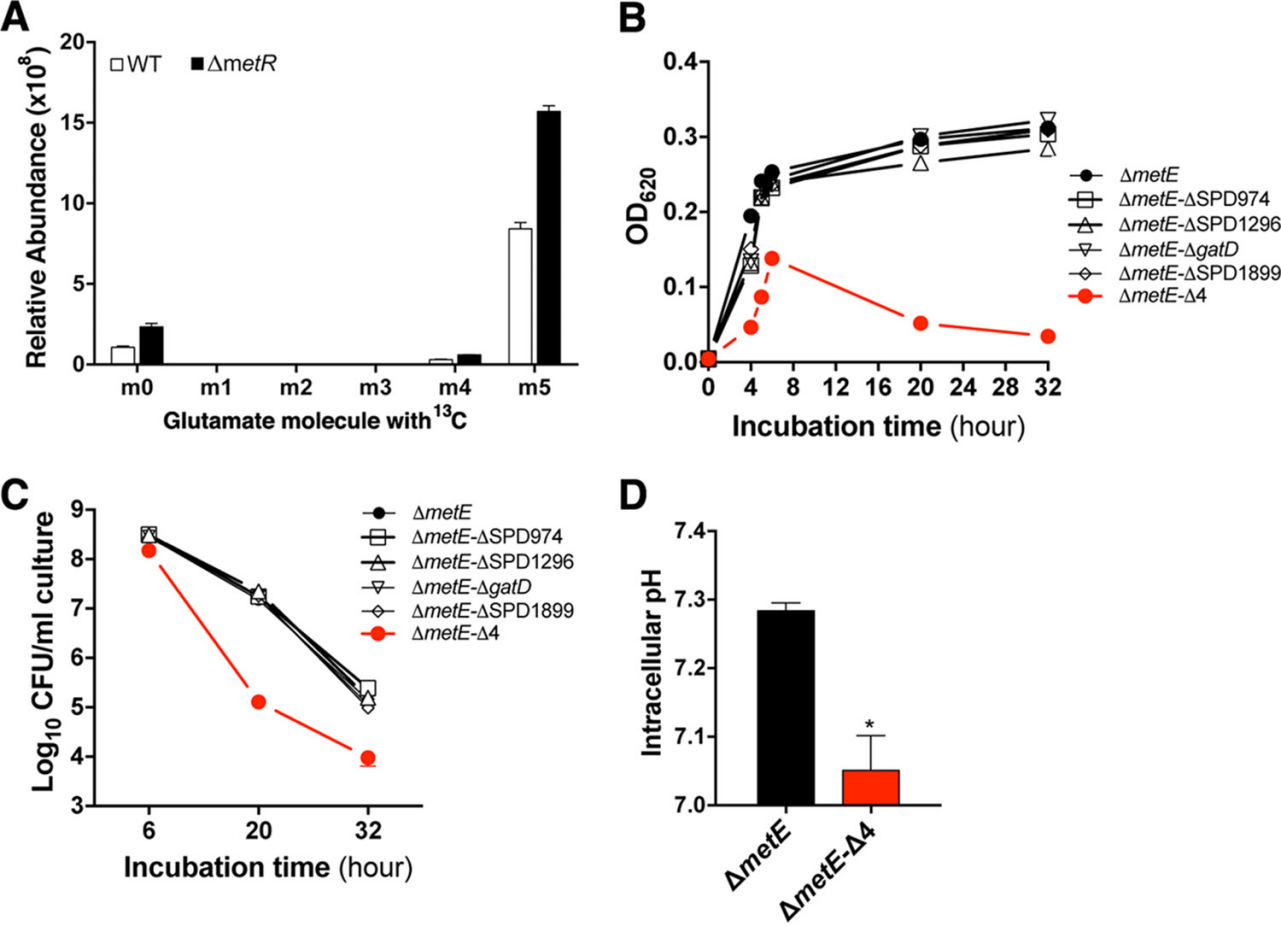
Requirement for glutamine deaminases for pneumococcal survival under methionine starvation. (A) Active conversion of glutamine to glutamic acid in S. pneumoniae under methionine starvation. D39 and the Δ*metR* mutant were grown in CDM with 1 μg/mL methionine and 100 μg/mL [^13^C_5_]glutamine for 6 h before measurement of the relative abundance of intracellular glutamic acid without (m0) or with 1 to 5 (m1 to m5) ^13^C-labeled carbon atoms. The data are presented as the percentages of each glutamate species of the total amino acids. (B) Growth of the glutamine deaminase gene mutants under methionine starvation. The Δ*metE* mutant and its derivatives lacking one or all (Δ*metE*-Δ4) of the *gatD*, SPD974, SPD1296, and SPD1899 genes were cultivated in CDM with 1 μg/mL methionine. Growth was measured and is presented as described in the legend of Fig. 1A. (C) Survival of the glutamine deaminase gene mutants under methionine starvation. Viable pneumococci in the cultures of the Δ*metE* derivatives from panel B were determined and are presented as described in the legend of Fig. 1B. (D) Cellular pH of the glutamine deaminase gene mutants under methionine starvation. The cultures of the Δ*metE* derivatives from panel B were used to determine the cellular pH, and the values are presented as described in the legend of Fig. 4C.

It is known that glutamine neutralizes the cellular pH by the deaminase-catalyzed conversion of glutamine to glutamic acid and the release of ammonium (38). Unlike glutamine, S. pneumoniae was previously shown to synthesize glutamic acid since it is one of the nonessential amino acids (14, 15). We first tested if glutamine deamination occurs under methionine starvation by measuring glutamate using [^13^C_5_]glutamine as a tracer. The vast majority of glutamate in the WT (89.1%) and the Δ*metR* mutant (87.5%) carried ^13^C at all 5 carbon atoms when cultivated under methionine starvation for 6 h (Fig. 7A, m5), thus indicating the conversion of glutamine to glutamate by deamination. In keeping with the massive accumulation of glutamine in the Δ*metE* and Δ*metR* mutants (Fig. 2), the level of [^13^C_5_]glutamate in the Δ*metR* mutant was 2 times higher than that in the WT, which is indicative of enhanced glutamine deamination under methionine starvation.

GatD (SPD1417) is the only pneumococcal glutamine deaminase that has been indicated to participate in cell wall peptidoglycan synthesis (41). In addition, there are 3 putative glutamine deaminases (SPD974, SPD1296, and SPD1899) in the D39 genome. We determined the functional contribution of these loci to pneumococcal survival under methionine starvation by deleting each gene or all 4 genes in the Δ*metE* mutant. While single-gene deletions did not significantly affect bacterial growth, the simultaneous removal of *gatD* and the other 3 putative deaminase genes led to a severe growth defect and autolysis (Fig. 7B), indicating functional redundancy among these genes. In a similar manner, all of the single-gene mutants displayed patterns of CFU counts similar to that of the Δ*metE* mutant at various time points at the stationary phase, and the mutant lacking all four genes showed a significant loss of CFU compared with the Δ*metE* mutant (Fig. 7C). In agreement with the function of glutamine deaminases in acid resistance in other bacteria (38, 42), the Δ*metE-*Δ4 mutant showed significantly lower cellular pH than the Δ*metE* mutant (Fig. 7D). Taken together, these data strongly suggested that multiple glutamine deaminases are involved in the maintenance of a prosurvival cellular pH by converting glutamine to glutamic acid.

### Glutamine accumulation occurs broadly in response to the shortage of other amino acids.

**FIG 8:**
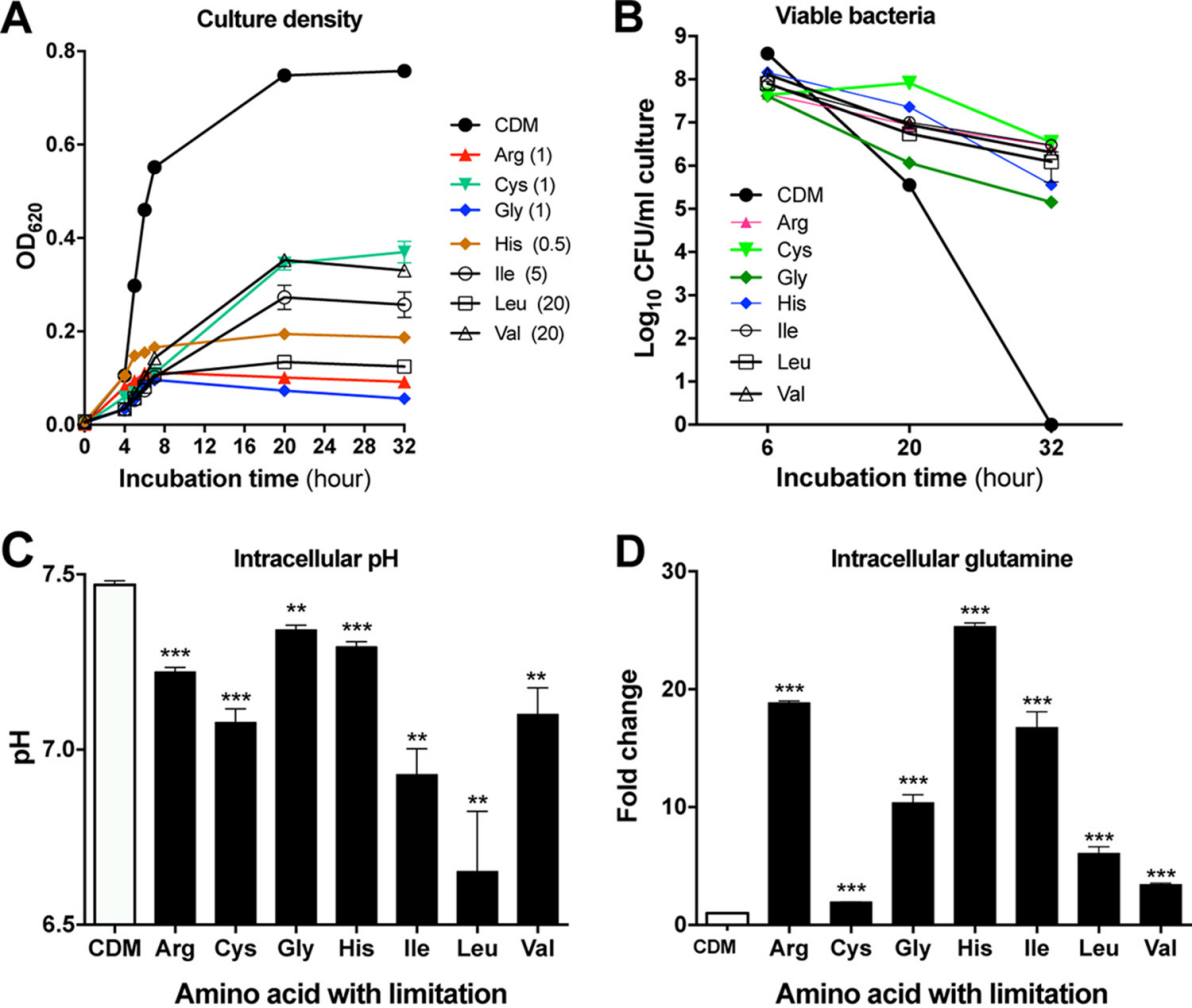
Enhanced pneumococcal survival under limitation of other essential amino acids. (A) Growth kinetics of S. pneumoniae D39 under starvation of 7 other essential amino acids. Pneumococci were cultivated in CDM with a reduced concentration of arginine (1 μg/ml), cysteine (1 μg/ml), glycine (1 μg/ml), histidine (0.5 μg/ml), isoleucine (5 μg/ml), leucine (20 μg/ml), or valine (20 μg/ml). The final concentration of each amino acid is indicated at the right of its abbreviation. (B) Survival of D39 under starvation of 7 other essential amino acids. The cells were grown in cultures as described for panel A. The viable bacteria were quantified as CFU at various time points. (C and D) Cellular pH (C) and intracellular glutamine (D) of D39 under starvation of seven other essential amino acids. The cells were grown in cultures as described for panel A. Measurements of cellular pH and glutamine level were carried out at 6 or 20 h when starvation occurred.

To assess if the glutamine-mediated prosurvival state also occurs upon the shortage of other amino acids, we characterized the survival, intracellular glutamine concentration, and cellular pH of D39 cells that were cultivated in CDM with a limited supply of arginine, cysteine, glycine, histidine, isoleucine, leucine, or valine, the 7 amino acids essential for D39 growth (besides glutamine) (14, 15). We initially tested the level of sensitivity of D39 to the shortage of these amino acids by growing the bacterium in CDM with various concentrations of single amino acids. The results showed that S. pneumoniae cells are more sensitive to the limitation of several amino acids (isoleucine, leucine, and valine) than to the limitation of the others (arginine, cysteine, glycine, and histidine) in growth perturbation (Fig. S4). We thus selected the appropriate concentrations of these amino acids at which bacterial growth was severely inhibited for the starvation experiments (Fig. 8A). In keeping with the methionine starvation-enhanced bacterial survival, pneumococci in the medium with a shortage of the individual essential amino acids also showed higher CFU counts at stationary phase (e.g., 20 and 32 h) than their counterparts cultivated in standard CDM (Fig. 8B). This observation indicated that amino acid starvation broadly enhances pneumococcal survival.

10.1128/msphere.00625-22.4 FIG S4 Growth and survival of S. pneumoniae D39 under essential amino acid starvation. (A to D) Growth curves (OD_620_) of pneumococcal D39 under starvation of different amino acids. Bacteria were grown in CDM with histidine concentrations of 0.1, 0.2, 0.5, 1, and 320 μg/ml (A), isoleucine concentrations of 1, 5, 10, 20, and 200 μg/ml (B), leucine concentrations of 1, 5, 10, 20, and 200 μg/ml (C), and valine concentrations of 1, 5, 10, 20, and 200 μg/ml (D). (E to H) Survival (CFU) of pneumococcal D39 under starvation of different amino acids. Bacteria were grown in CDM with histidine concentrations of 0.1, 0.2, 0.5, 1, and 320 μg/ml (E), isoleucine concentrations of 1, 5, 10, 20, and 200 μg/ml (F), leucine concentrations of 20, and 200 μg/ml (G), and valine concentrations of 20 and 200 μg/ml (H). Viable pneumococci of the cultures were quantified by CFU per milliliter. Download FIG S4, TIF file, 0.7 MB. Copyright © 2023 Zhang et al. 2023 Zhang et al. https://creativecommons.org/licenses/by/4.0/ This content is distributed under the terms of the Creative Commons Attribution 4.0 International license.

To determine if amino acid starvation-enhanced bacterial survival is associated with a prosurvival cellular pH, we characterized the cellular acidity of pneumococci cultured with single limitations of the 7 essential amino acids. This experiment revealed a moderate but significant reduction in the cellular pH of pneumococci cultivated in CDM with individual shortages of the 7 amino acids, even though the levels of the impact varied among the nutrients (Fig. 8C). This result suggested that amino acid deficiency enhances pneumococcal survival by adopting a prosurvival intracellular pH.

Finally, we assessed the involvement of intracellular glutamine in amino acid starvation-enhanced survival. The abundance of intracellular glutamine was significantly increased in pneumococci cultivated in amino-acid-deficient CDM, although the levels of glutamine accumulation varied for each amino acid (Fig. 8D). Histidine showed the most dramatic impact on the intracellular glutamine concentration, with an increase of 24.3-fold. Shortages of arginine (by 17.8-fold), isoleucine (by 15.8-fold), and glycine (by 9.4-fold) resulted in intermediate enhancements of the intracellular glutamine concentration. Although cysteine starvation yielded the highest levels of pneumococcal survival (Fig. 8B), this condition generated the lowest increase in the intracellular glutamine concentration, suggesting that cysteine starvation induces an additional adaptation response beyond glutamine accumulation. Together, these results have prompted us to conclude that amino acid starvation broadly induces a prosurvival metabolic state in S. pneumoniae, which is characterized by the massive accumulation of glutamine and lactate and a lower intracellular pH.

## DISCUSSION

It has been well characterized that amino acid starvation induces a stringent response by producing the (pp)pGpp alarmone nucleotides in many bacteria (3, 5). However, it is largely unknown how bacteria adapt to amino acid starvation at the metabolic level. This study has systematically characterized the metabolomic changes in S. pneumoniae under methionine starvation. To the best of our knowledge, this is the first study to systematically characterize the bacterial metabolome under amino acid starvation. Our data have uncovered a new metabolic mechanism of the stringent response in which S. pneumoniae assimilates glutamine and lactate to form a prosurvival metabolic state with a lower intracellular pH, which inhibits bacterial growth for prolonged survival.

Consistent with the function of the stringent response in bacterial adaptation (5, 43), our data have demonstrated that amino acid starvation prolongs the survival of pneumococci. Pneumococci under the limitation of methionine or 7 essential amino acids grew to a lower culture density before entering the stationary phase, but they maintained much higher levels of viability in the stationary phase. The RSH protein Rel_spn_ of S. pneumoniae has been shown to produce ppGpp and pppGpp, particularly after treatment with mupirocin, an inducer of the stringent response (15). In agreement with the role of the stringent response in bacterial survival, Rel_spn_ is not required for bacterial growth in complex medium but is essential for growth in CDM (15). Interestingly, natural allelic variations in Rel_spn_ have been reported to affect pneumococcal resistance to neutrophil killing *in vitro* and nasal colonization in mice (44). Although we were unable to determine if Rel_spn_ is involved in prolonged pneumococcal survival under methionine starvation due to the requirement for Rel_spn_ for pneumococcal growth in CDM (15), we believe that prolonged survival under the limitation of methionine and the other essential amino acids depends on the regulation of Rel_spn_, as described previously for many other bacteria (4).

Metabolomic profiling revealed dramatic changes in the metabolism of methionine-starved pneumococci. We detected a number of metabolites whose levels were decreased to various extents, particularly the derivatives of methionine, nucleotides, and lipids, which apparently represented downshifts in RNA synthesis and lipid metabolism under methionine starvation, characteristics of the stringent response. On the other end, methionine starvation induced the accumulation of many metabolites. The striking increase in cAMP indicates the importance of this signaling molecule in the cellular response to methionine limitation. cAMP has been shown to enhance the production of (p)ppGpp by interacting with cAMP receptor protein (CRP) under glucose starvation (29), but its contribution to the bacterial response to amino acid starvation is unknown. In the context of substantial variations in lipid metabolites, the dramatic accumulation of mevalonate in methionine-starved pneumococci may be the result of reduced lipid metabolism in the stringent response. Alternatively, it is possible that mevalonate builds up due to a metabolic gap(s) in the methionine-associated activity of the mevalonate pathway. The mevalonate pathway is highly conserved in all bacteria for the generation of isoprenoid compounds for many cellular functions such as cell wall synthesis, capsule synthesis, membrane integrity, electron transport, and protein modification (31). The mevalonate pathway in S. pneumoniae is essential for *in vitro* growth (32) and cell division (33).

It was surprising to observe *N*-acetylglutamine and l-glutamine as 2 of the top 5 metabolites enriched in the Δ*metE* mutant. *N*-Acetylglutamine (or aceglutamide) is a neuroprotective drug in humans (45), but its biological activity is poorly characterized in both eukaryotes and prokaryotes. Due to the overwhelming abundance of intracellular glutamine over *N*-acetylglutamine, *N*-acetylglutamine may represent metabolic spillover from glutamine. The major changes in the metabolome of the Δ*metE* mutant under methionine starvation have been confirmed in the methionine synthesis-deficient Δ*metR* mutant. The top 10 metabolites in the decreased and increased categories are virtually identical between the metabolomes of the two mutants (see Tables S1 and S2 in the supplemental material). Moreover, these metabolomic changes became undetectable when sufficient methionine was added to CDM. These results have demonstrated the reliability of the metabolomic approach for understanding the stringent response and perhaps other stress responses.

This work has shown that S. pneumoniae adapts to methionine starvation by adopting a prosurvival metabolic state with a lower intracellular pH. The pneumococci under methionine starvation (e.g., the Δ*metE* mutant with 1 μg/mL methionine) showed an intracellular pH of 7.24, compared with a pH of 7.59 for the cells grown in methionine-sufficient medium (e.g., the Δ*metE* mutant with 1 μg/mL methionine). The lactate supplementation experiment showed that modest changes in the intracellular pH can have a great impact on the viability of S. pneumoniae cells in the stationary phase. Pneumococci with an intracellular pH of 7.2 showed optimal survival, but their counterparts with intracellular pHs of 7.6 and 6.9 displayed 29- and 59-fold reductions in CFU counts, respectively. While this study is the first to link intracellular pH homeostasis to bacterial survival under nutrient deprivation, relatively lower intracellular pHs have been shown to promote bacterial survival against antibiotic toxicity. Goode et al. recently reported that antibiotic-tolerant E. coli persister cells possess a lower intracellular pH than their susceptible counterparts, which is maintained by a tryptophanase-dependent mechanism (46). In Mycobacterium smegmatis, the control of cellular pH homeostasis is associated with bacterial survival against antibiotic treatment (47). Although it remains to be defined how a lower pH precisely enhances bacterial survival against nutrient starvation and antibiotic toxicity, it is conceivable that cytoplasmic acidification may inhibit the optimal activities of metabolic enzymes and thereby halt cellular metabolism, which has been recognized as a general mechanism of bacterial tolerance to antibiotics (48). In Saccharomyces cerevisiae, glucose starvation leads to coincidental reductions in the mitochondrial pH and growth, indicative of reduced pH as a subcellular state to control metabolism and growth (49).

The remarkable enrichment of lactate in methionine-starved pneumococci suggests that it serves as a major driver of intracellular pH reduction. Lactate was enriched 2-fold in the metabolome of methionine-starved pneumococci. Since lactate is a well-known cytoplasmic proton donor (40, 50), it is reasonable to believe that the increase in the cellular lactate concentration leads to a reduction in the pH. The intracellular enrichment of lactate appears to be the result of enhanced glycolysis under methionine starvation. Except for 1,3-bisphosphoglycerate, all components of the glycolysis pathway were detected in S. pneumoniae and enriched under methionine starvation. The most enriched metabolites are glyceraldehyde 3-phosphate, phosphoenolpyruvate, and lactate. The enhanced glycolysis in methionine-starved pneumococci fully agrees with the dramatic increase in cAMP in the metabolome. Meyer et al. have reported that cAMP enhances sugar metabolism in E. coli during the stringent response (29). In contrast, many components of the tricarboxylic acid (TCA) cycle were not detected in S. pneumoniae. Only citrate, α-ketoglutarate (α-KG), succinate, and fumarate were detected in S. pneumoniae. Due to the lack of a respiratory electron transport chain, S. pneumoniae can perform anaerobic respiration only (51). Besides, S. pneumoniae does not have a complete TCA cycle (51). Fermentation is the major way for S. pneumoniae to generate ATP. S. pneumoniae can utilize over 30 carbohydrates. Glucose and other carbohydrates can be converted to pyruvate by glycolysis. Pyruvate is primarily converted to lactate by lactate dehydrogenase (LDH), generating NAD^+^ necessary for glycolysis. Pyruvate can also be catabolized to some other metabolites; for example, pyruvate formate lyase (PFL) catabolizes pyruvate into formate and acetyl-CoA. Acetyl-CoA can be converted to acetate, generating ATP (51). In glucose fermentation, pyruvate is primarily converted to lactate by LDH (52). In galactose fermentation, pyruvate is primarily converted to acetyl-CoA and formate by PFL (53).

Our data strongly suggested that glutamine enhances pneumococcal survival by balancing the intracellular pH under methionine starvation. As one of the most enriched molecules in methionine-starved S. pneumoniae, intracellular glutamine accumulated along with the reduction in the intracellular pH. Moreover, the intracellular glutamine and pH were returned to their physiological levels under methionine-sufficient conditions. Since glutamine is known to enhance E. coli acid resistance by neutralizing the intracellular pH via the enzymatic release of ammonia and glutamic acid (38), it is reasonable to envision that the ammonia from glutamine deamination serves as a major metabolite to neutralize the protons released from lactate and other acidic metabolites. Consistent with the role of glutamine deamination in neutralizing the intracellular pH, the Δ*metE* strain lacking the glutamine deaminase genes displayed a low cellular pH and impaired survival. Furthermore, glutamic acid (the product of glutamine deamination) (38), along with its 7 derivatives, was abundantly enriched in the metabolomes of methionine-starved Δ*metE* and Δ*metR* cells. In short, we postulate the following conceptual model: the fine orchestration of the major proton producer (e.g., lactate) and neutralizer (e.g., glutamine) in methionine-starved bacteria forms a prosurvival metabolic state with a lower pH under methionine starvation and perhaps other stress conditions (see below), which in turn slows cellular metabolism and thereby promotes bacterial survival. The “yin and yang” roles of lactate and glutamine in balancing the intracellular pH under methionine starvation are supported by our observation that the addition of lactate to stationary-phase cultures of D39 resulted in the significant assimilation of glutamine, suggesting that glutamine is accumulated in response to the reduction in the cellular pH.

The glutamine-mediated survival of methionine-starved pneumococci is reminiscent of a similar finding in penicillin-treated S. pneumoniae (54). Pneumococci exposed to penicillin accumulate glutamine and glutamate by approximately 40- and 6-fold, respectively, which are similar to the levels of the two amino acids in the methionine-starved counterpart. Moreover, the addition of glutamine to the culture medium significantly enhances pneumococcal survival against the lethal action of penicillin. In light of these data, it is tempting to postulate that glutamine enhances pneumococcal survival under penicillin toxicity and methionine starvation conditions by a similar pH-balancing mechanism. First, pneumococci under the two stress conditions assimilate similar levels of intracellular glutamine and glutamic acid. Second, the addition of glutamine to the culture medium enhances pneumococcal survival under the two conditions. Finally, S. pneumoniae cells achieve massive glutamine accumulation by uptake from the extracellular milieu under penicillin exposure and methionine starvation conditions. El Khoury et al. suggested that penicillin-induced glutamine accumulation occurs by amino acid uptake since penicillin treatment inhibits the transcription of the *glnA* gene (54). *glnA* encodes a glutamine synthetase for the conversion of glutamate and ammonia to glutamine (36). Our isotope-tracking data have provided unequivocal evidence that S. pneumoniae mainly acquires glutamine from the medium under methionine starvation, although the precise mechanism remains to be determined. These findings agree with previous reports of glutamine as an essential amino acid in this bacterium (14, 15).

Multiple lines of evidence suggest that S. pneumoniae adopts the prosurvival metabolic state under shortages of other amino acids beyond methionine. To various extents, S. pneumoniae unanimously displayed prolonged survival in the stationary phase with individual shortages of the seven essential amino acids. In the meantime, the bacteria showed lower intracellular pHs and significant glutamine accumulation. It should be noted that limitations of different amino acids resulted in variable impacts on the intracellular pH and glutamine. These phenotypic variations might be caused by different extents of stress imposed by the concentrations of the amino acids. Alternatively, shortages of individual amino acids can result in variable metabolic impacts due to their functional differences. Glutamine has also been found to be preferentially accumulated in Erwinia chrysanthemi at high salt concentrations, which is considered to promote bacterial tolerance to osmotic stress (55). Together with the findings of El Khoury et al. (54), our data strongly suggest that glutamine accumulation enhances pneumococcal survival against amino acid starvation by balancing the intracellular pH, although our data cannot exclude the possible role of glutamine in osmotic balance. Taken together, these findings indicate that glutamine accumulation represents a metabolomic mechanism for pneumococcal adaptation to a wide range of stress conditions.

It has been well documented that S. pneumoniae is able to adapt to nutrient starvation and antibiotic treatment. Walsh and Camilli found that the bacterium can survive and remain infectious for a long time under dehydration conditions (56). Our recent work shows that S. pneumoniae colonizes the host’s upper airway with poor availability of methionine (24). A large body of literature indicated the remarkable resilience of S. pneumoniae against antibiotic treatment in children with acute otitis media (57), as manifested by the reisolation of the same strains in different otitis media episodes in the same children (58, 59). In this context, understanding the mechanisms of stress adaptation by S. pneumoniae is of great importance for improving therapeutic strategies and developing new antimicrobials. The glutamine-based stress adaptation mechanism may be targeted to eliminate residual pneumococci in human infections.

## MATERIALS AND METHODS

### Bacterial cultivation and reagents.

S. pneumoniae serotype 2 strain D39 (60) was used as a parental strain in this study. Pneumococci were cultured in Todd-Hewitt broth supplemented with 0.5% yeast extract (THY broth), chemically defined medium (CDM), or tryptic soy agar (TSA) plates with sheep blood (3%) at 37°C with 5% CO_2_, as previously described (61). CDM was prepared according to methods described in a previous study (34). The media were supplemented with the appropriate antibiotics when necessary, as described previously (61). All chemicals and enzymes for molecular biology were products from Sigma (Beijing, China) and New England BioLabs (Beijing, China), respectively. The strains used in this study are described in Table 2.

**TABLE 2:** Bacterial strains used in this study

Strain	Description	Reference
D39	Streptococcus pneumoniae serotype 2; encapsulated	60
TH4306	D39 derivative; *rpsL1*	61
TH9197	TH4306 Δ*metR*	24
TH9660	TH4306 Δ*metE*	24
TH16194	TH9660 SPD1098(F277Y,S335T,S384A); F277, S335, and S384 of SPD1098 were mutated (F277Y, S335T, and S384A) in TH9660	This study
TH16216	TH9660 Δ*lytA*; *lytA* was removed from TH9660	This study
TH16219	TH9660 ΔSPD974; SPD974 was removed from TH9660	This study
TH16220	TH9660 ΔSPD1296; SPD1296 was removed from TH9660	This study
TH16221	TH9660 Δ*gatD*; *gatD* was removed from TH9660	This study
TH16223	TH9660 ΔSPD1899; SPD1899 was removed from TH9660	This study
TH16224	TH9660 Δ*gatD* ΔSPD1899 ΔSPD0974 ΔSPD1296; *gatD*, SPD1899, SPD0974, and SPD1296 were sequentially removed from TH9660	This study
TH16225	TH9660::pIB166-pH-GFP; plasmid pIB166 with pH-GFP was transformed into TH9660	This study
TH16226	TH9197::pIB166-pH-GFP; plasmid pIB166 with pH-GFP was transformed into TH9197	This study
TH16227	D39::pIB166-pH-GFP; plasmid pIB166 with pH-GFP was transformed into TH4306	This study
TH16228	TH16224::pIB166-pH-GFP; plasmid pIB166 with pH-GFP was transformed into TH16224	This study
TH16308	E. coli BL21 derivative; SBD(25–264) of SPD0412 with an N-terminal His_6_ tag was inserted into plasmid pET28a to generate plasmid pTH16308, and pTH16308 was transformed into E. coli BL21 to generate TH16308	This study
TH16309	E. coli BL21 derivative; SBD(26–264) of SPD0530 with an N-terminal His_6_ tag was inserted into plasmid pET28a to generate plasmid pTH16309, and pTH16309 was transformed into E. coli BL21 to generate TH16309	This study
TH16310	E. coli BL21 derivative; SBD1(24–247) of SPD1098 with an N-terminal His_6_ tag was inserted into plasmid pET28a to generate plasmid pTH16310, and pTH16310 was transformed into E. coli BL21 to generate TH16310	This study
TH16311	E. coli BL21 derivative; SBD2(262–488) of SPD1098 with an N-terminal His_6_ tag was inserted into plasmid pET28a to generate plasmid pTH16311, and pTH16311 was transformed into E. coli BL21 to generate TH16311	This study
TH16312	E. coli BL21 derivative; SBD(26–278) of SPD1328 with an N-terminal His_6_ tag was inserted into plasmid pET28a to generate plasmid pTH16312, and pTH16312 was transformed into E. coli BL21 to generate TH16312	This study

### Mutant construction.

All gene deletion mutants were constructed from strain TH4306, a streptomycin-resistant derivative of strain D39, by natural transformation using Janus cassette (JC)-based counterselection, as previously described (62, 63). Briefly, the up- and downstream sequences of the target genes and JC were individually amplified for the purpose of gene replacement by the JC, which contains the kanamycin resistance gene *kan* for selection and *rpsL* for counterselection. The amplicons were sequentially linked by enzymatic digestion and ligation or fusion PCR before being used for natural transformation. The transformants were selected by kanamycin resistance. For counterselection, the flanking regions of the target genes were amplified and fused by digestion with BsaI as described previously (62). The primers used in this work are listed in Table S5 in the supplemental material. The specific setup for the construction of each mutant is described in Table S6.

10.1128/msphere.00625-22.9 TABLE S5 Primers used in this study. Download Table S5, DOCX file, 0.02 MB. Copyright © 2023 Zhang et al. 2023 Zhang et al. https://creativecommons.org/licenses/by/4.0/ This content is distributed under the terms of the Creative Commons Attribution 4.0 International license.

10.1128/msphere.00625-22.10 TABLE S6 PCR amplifications used for pneumococcal mutagenesis in this study. Download Table S6, DOCX file, 0.01 MB. Copyright © 2023 Zhang et al. 2023 Zhang et al. https://creativecommons.org/licenses/by/4.0/ This content is distributed under the terms of the Creative Commons Attribution 4.0 International license.

### Characterization of bacterial growth and survival.

The growth of pneumococci was characterized essentially as previously described (23). Briefly, bacteria were grown in THY broth to an optical density at 620 nm (OD_620_) of 0.5, washed twice with Ringer’s solution by centrifugation and resuspension, resuspended in Ringer’s solution to an OD_620_ of 0.5, and diluted at a 1:100 ratio in standard CDM (34) or CDM with various modifications of the amino acid content. Bacterial growth was monitored by measuring the OD_620_.

Quantification of bacterial survival was carried out by plating original cultures and/or their dilutions onto TSA blood plates to enumerate the CFU. Each CFU was regarded as a single viable pneumococcus for the purpose of data analysis.

### Cellular pH determination.

The cellular pH was determined using a pH-sensitive green fluorescent protein (pH-GFP) as previously described (39, 64), according to principles established previously (65). Briefly, plasmid pIB166 harboring the pH-GFP gene was transformed into pneumococci by natural transformation. Strains with the pH-GFP plasmid were cultured in THY broth until mid-log phase (OD_620_ of 0.4), washed twice with fresh CDM by centrifugation and resuspension, and diluted into fresh CDM. At the time of pH detection, bacteria at an OD_620_ of 0.4 were collected and washed once with colorless CDM, which excludes glutamine, glutamate, methionine, cysteine, cystine, lysine, Fe_2_SO_4_·7H_2_O, MnSO_4_·4H_2_O, and vitamins (66). Bacterial pellets were resuspended in 800 μL colorless CDM and dispensed into black 96-well plates (200 μL/well) (Corning Incorporated, USA). Fluorescence was determined at reading 1 (excitation at 395 nm and emission at 510 nm) and reading 2 (excitation at 475 nm and emission at 510 nm) by using an Infinite M Plex instrument (Tecan, Switzerland). The ratio of “reading 1 − blank 1” to “reading 2 − blank 2” (*Y*) was used to determine the cellular pH value (*X*) with the following equation: *X* = {6.53 − log_10_[1.7453/(*Y* − 0.4027) − 1]}/0.9127 (39).

### Metabolomics analysis.

Metabolomics analysis was carried out as previously described (67), with minor modifications. Six hours after inoculation in CDM, bacteria at an OD_620_ of 15 were collected and washed twice with ice-cold Ringer’s solution by centrifugation and resuspension in a Sorvall RC-6 plus high-speed centrifuge at 4°C at 22,040 × *g* for 10 min. After being frozen in liquid nitrogen, bacterial pellets were resuspended in 1 mL 80% methanol stored at −80°C. The solution was transferred into a 2-mL grinding tube containing 1 g of glass beads (0.4 to 0.6 mm, catalog number BE6098-100g; Easybio, China) and ground using a Bead Ruptor 12 instrument (Omni International, USA) at high speed (1 min each time) 10 times, with a 1-min interruption for sample cooling in ice water between grindings. Ground samples were cooled at −80°C for 1 h and centrifuged at 4°C at 12,000 rpm in a microcentrifuge for 20 min. The supernatants were collected and dried for metabolomics analysis in a vacuum dryer.

Metabolomic analysis was performed using the Q Exactive mass spectrometer (Thermo, USA) coupled with an Ultimate 3000 liquid chromatography system (Thermo, USA). An Acquity ultraperformance liquid chromatography (UPLC) ethylene-bridged hybrid (BEH) amide column (2.1 by 100 mm, 1.7 μm; Waters) was applied for analysis in positive-ion mode. Mobile phase A was prepared using 7.5 mM ammonium formate dissolved in 5:95 (vol/vol) H_2_O-acetonitrile (ACN) with 0.001% formic acid. Mobile phase B contained 7.5 mM ammonium formate dissolved in 50:50 (vol/vol) H_2_O-ACN with 0.001% formic acid. An Acquity UPLC BEH C_18_ column (2.1 by 100 mm, 1.7 μm; Waters) was incorporated for analysis in negative-ion mode. Mobile phases A and B contained 5 mM ammonium bicarbonate (aqueous) and acetonitrile, respectively. Resolutions of 70,000 and 35,000 were applied for the precursor scan and fragment scan, respectively. Data-dependent acquisition with the top 10 most intense precursors selected for fragmentation was used for the analysis. Metabolite identification relied on an in-house library containing fragment spectra of ~1,500 metabolites. Two levels of identification were included in the results. One was confirmed by fragment matching in the library, and the other was assigned based on precursor ion masses. Chromatographic areas were used for quantitation. Metabolites with significant changes were calculated based on Student’s *t* test (*P* < 0.05) and abundance changes (>50%).

### Amino acid quantification.

Quantification of intracellular methionine and glutamine was accomplished by the separation and identification of individual metabolites using liquid chromatography and mass spectrometry. Specifically, metabolites were extracted from bacteria at an OD_620_ of 0.5 as described above for the metabolomic analysis. The supernatants in 80% methanol were analyzed directly without drying. A 6500 plus QTrap mass spectrometer (AB Sciex, USA) coupled with an Acquity UPLC H-class system (Waters, USA) was used for metabolite quantitation. Chromatographic separation was achieved using an Acquity UPLC BEH amide column (2.1 by 100 mm, 1.7 μm; Waters). Mobile phase A contained 5:95 (vol/vol) high-performance liquid chromatography (HPLC)-grade H_2_O-ACN with 7.5 mM ammonium formate, and mobile phase B contained 50:50 (vol/vol) H_2_O-ACN with 7.5 mM ammonium formate. Data were acquired in multiple-reaction monitoring (MRM) mode in positive mode. The ion transitions were optimized using chemical standards. The nebulizer gas (gas 1), heater gas (gas 2), and curtain gas were set at 55, 55, and 30 lb/in^2^, respectively. The ion spray voltage was 5,500 V for positive-ion mode. The optimal probe temperature was determined to be 550°C, and the column oven temperature was set at 45°C. SCIEX OS 1.6 software (AB Sciex, USA) was applied for metabolite identification and peak integration.

### Protein expression and purification.

The expression and purification of recombinant pneumococcal substrate binding domains (SBDs) was achieved in E. coli BL21 as described previously (68). Briefly, the SBDs of SPD0412, SPD0530, SPD1098 (SBD1 and SBD2), and SPD1328 were amplified from the D39 genome using primer pairs Pr18539/Pr18540, Pr18541/Pr18542, Pr18547/Pr18548, Pr18545/Pr18546, and Pr18543/Pr18544, respectively, and cloned into the NcoI/BamHI sites of pET28a. The sequence encoding an N-terminal His_6_ tag was added in the 5’ primers of each construct. Recombinant proteins were affinity purified with nickel-Sepharose resin columns.

### Isothermal titration calorimetry.

ITC experiments were performed using the MicroCal iTC_200_ system at 25°C as described previously (17). In brief, amino acid ligands were dissolved in ITC buffer at a concentration of 500 to 1,000 μM. SBD proteins were concentrated to 30 to 100 μM using a Millipore 3-kDa-molecular-weight-cutoff (MWCO) filter (Millipore, USA). The ligands in a syringe were added stepwise to SBD proteins in an ITC cell with a stirring speed of 1,000 rpm. Heat transfer (microcalories per second) measured from injections was analyzed to determine the dissociation constant (*K_D_*) using nonlinear regression fitting of a single-binding-site model (MicroCal ORIGIN software, OriginLab, USA).

### Isotope tracing.

Isotope tracing of [^13^C_5_]glutamine in pneumococcal cells was carried out by bacterial sample preparation as described above for the metabolomic analysis, with the exception that glutamine in CDM was replaced by the stable isotope [^13^C_5_]glutamine. Isotope-labeled metabolites were analyzed using a QE mass spectrometer with the Ultimate 3000 system (Thermo, USA). Analysis was performed in positive-ion mode. An Acquity UPLC BEH amide column (2.1 by 100 mm, 1.7 μm; Waters) was used for chromatographic separation. In this method, 5:95 (vol/vol) H_2_O-ACN containing 7.5 mM ammonium formate with 0.001% formic acid was used as mobile phase A, and 50:50 (vol/vol) H_2_O-ACN containing 7.5 mM ammonium formate with 0.001% formic acid was used as mobile phase B. Resolutions of 70,000 were applied for MS scans. Tracefinder 3.2 (Thermo, USA) was used for data analysis with simulated mass-to-charge ratios of isotope-labeled metabolites. Chromatographic areas were used for quantitation.

### Statistical analysis.

All experiments reported in this work were conducted with triplicate samples and repeated at least once, except for the metabolomic analyses of the *metE* and *metR* mutants (with two technical repeats). The relevant data are presented as means ± SEM (standard errors of means) and were analyzed by two-tailed unpaired Student’s *t* test in GraphPad Prism 7 for Mac OS X. Significant differences are defined by *P* values of <0.05 (indicated by * in the figures), <0.01 (**), <0.001 (***), and <0.0001 (****).

## References

[1] Potrykus K, Cashel M. 2008. (p)ppGpp: still magical? Annu Rev Microbiol 62:35–51. doi:10.1146/annurev.micro.62.081307.162903. 18454629

[2] Cashel M, Gallant J. 1969. Two compounds implicated in the function of the RC gene of Escherichia coli. Nature 221:838–841. doi:10.1038/221838a0. 4885263

[3] Haseltine WA, Block R. 1973. Synthesis of guanosine tetra- and pentaphosphate requires the presence of a codon-specific, uncharged transfer ribonucleic acid in the acceptor site of ribosomes. Proc Natl Acad Sci USA 70:1564–1568. doi:10.1073/pnas.70.5.1564. 4576025 PMC433543

[4] Atkinson GC, Tenson T, Hauryliuk V. 2011. The RelA/SpoT homolog (RSH) superfamily: distribution and functional evolution of ppGpp synthetases and hydrolases across the tree of life. PLoS One 6:e23479. doi:10.1371/journal.pone.0023479. 21858139 PMC3153485

[5] Irving SE, Choudhury NR, Corrigan RM. 2021. The stringent response and physiological roles of (pp)pGpp in bacteria. Nat Rev Microbiol 19:256–271. doi:10.1038/s41579-020-00470-y. 33149273

[6] Aggarwal SD, Lloyd AJ, Yerneni SS, Narciso AR, Shepherd J, Roper DI, Dowson CG, Filipe SR, Hiller NL. 2021. A molecular link between cell wall biosynthesis, translation fidelity, and stringent response in *Streptococcus pneumoniae*. Proc Natl Acad Sci USA 118:e2018089118. doi:10.1073/pnas.2018089118. 33785594 PMC8040666

[7] Smallman TR, Williams GC, Harper M, Boyce JD. 2022. Genome-wide investigation of Pasteurella multocida identifies the stringent response as a negative regulator of hyaluronic acid capsule production. Microbiol Spectr 10:e00195-22. doi:10.1128/spectrum.00195-22. 35404102 PMC9045168

[8] O’Brien KL, Wolfson LJ, Watt JP, Henkle E, Deloria-Knoll M, McCall N, Lee E, Mulholland K, Levine OS, Cherian T, Hib and Pneumococcal Global Burden of Disease Study Team. 2009. Burden of disease caused by *Streptococcus pneumoniae* in children younger than 5 years: global estimates. Lancet 374:893–902. doi:10.1016/S0140-6736(09)61204-6. 19748398

[9] van der Poll T, Opal SM. 2009. Pathogenesis, treatment, and prevention of pneumococcal pneumonia. Lancet 374:1543–1556. doi:10.1016/S0140-6736(09)61114-4. 19880020

[10] Janoff EN, Musher DM. 2015. *Streptococcus pneumoniae*, p 2310–2327. *In* Bennett JE, Dolin RD, Blaser MJ (ed), Mandell, Douglas, and Bennett’s principles and practice of infectious diseases, 8th ed, vol 2. Elsevier Saunders, Philadelphia, PA.

[11] Siegel SJ, Weiser JN. 2015. Mechanisms of bacterial colonization of the respiratory tract. Annu Rev Microbiol 69:425–444. doi:10.1146/annurev-micro-091014-104209. 26488280 PMC4760621

[12] Pezzulo AA, Gutierrez J, Duschner KS, McConnell KS, Taft PJ, Ernst SE, Yahr TL, Rahmouni K, Klesney-Tait J, Stoltz DA, Zabner J. 2011. Glucose depletion in the airway surface liquid is essential for sterility of the airways. PLoS One 6:e16166. doi:10.1371/journal.pone.0016166. 21311590 PMC3029092

[13] Philips BJ, Meguer JX, Redman J, Baker EH. 2003. Factors determining the appearance of glucose in upper and lower respiratory tract secretions. Intensive Care Med 29:2204–2210. doi:10.1007/s00134-003-1961-2. 14647890

[14] Härtel T, Eylert E, Schulz C, Petruschka L, Gierok P, Grubmüller S, Lalk M, Eisenreich W, Hammerschmidt S. 2012. Characterization of central carbon metabolism of *Streptococcus pneumoniae* by isotopologue profiling. J Biol Chem 287:4260–4274. doi:10.1074/jbc.M111.304311. 22167202 PMC3281726

[15] Kazmierczak KM, Wayne KJ, Rechtsteiner A, Winkler ME. 2009. Roles of *relSpn* in stringent response, global regulation and virulence of serotype 2 *Streptococcus pneumoniae* D39. Mol Microbiol 72:590–611. doi:10.1111/j.1365-2958.2009.06669.x. 19426208 PMC2739083

[16] Tettelin H, Nelson KE, Paulsen IT, Eisen JA, Read TD, Peterson S, Heidelberg J, DeBoy RT, Haft DH, Dodson RJ, Durkin AS, Gwinn M, Kolonay JF, Nelson WC, Peterson JD, Umayam LA, White O, Salzberg SL, Lewis MR, Radune D, Holtzapple E, Khouri H, Wolf AM, Utterback TR, Hansen CL, McDonald LA, Feldblyum TV, Angiuoli S, Dickinson T, Hickey EK, Holt IE, Loftus BJ, Yang F, Smith HO, Venter JC, Dougherty BA, Morrison DA, Hollingshead SK, Fraser CM. 2001. Complete genome sequence of a virulent isolate of *Streptococcus pneumoniae*. Science 293:498–506. doi:10.1126/science.1061217. 11463916

[17] Fulyani F, Schuurman-Wolters GK, Zagar AV, Guskov A, Slotboom DJ, Poolman B. 2013. Functional diversity of tandem substrate-binding domains in ABC transporters from pathogenic bacteria. Structure 21:1879–1888. doi:10.1016/j.str.2013.07.020. 23994008

[18] Basavanna S, Chimalapati S, Maqbool A, Rubbo B, Yuste J, Wilson RJ, Hosie A, Ogunniyi AD, Paton JC, Thomas G, Brown JS. 2013. The effects of methionine acquisition and synthesis on *Streptococcus pneumoniae* growth and virulence. PLoS One 8:e49638. doi:10.1371/journal.pone.0049638. 23349662 PMC3551916

[19] Kloosterman TG, Kuipers OP. 2011. Regulation of arginine acquisition and virulence gene expression in the human pathogen *Streptococcus pneumoniae* by transcription regulators ArgR1 and AhrC. J Biol Chem 286:44594–44605. doi:10.1074/jbc.M111.295832. 22084243 PMC3248006

[20] Schulz C, Gierok P, Petruschka L, Lalk M, Mader U, Hammerschmidt S. 2014. Regulation of the arginine deiminase system by ArgR2 interferes with arginine metabolism and fitness of *Streptococcus pneumoniae*. mBio 5:e01858-14. doi:10.1128/mBio.01858-14. 25538192 PMC4278536

[21] Kozak M. 1983. Comparison of initiation of protein synthesis in procaryotes, eucaryotes, and organelles. Microbiol Rev 47:1–45. doi:10.1128/mr.47.1.1-45.1983. 6343825 PMC281560

[22] Zhang J-R, Marinus MG, Deng H. 2019. Methylation and other modifications of nucleic acids and proteins, p 83–92. *In* Schmidt TS (ed), Encyclopedia of microbiology, 4th ed. Elsevier, Amsterdam, Netherlands.

[23] Shelver D, Rajagopal L, Harris TO, Rubens CE. 2003. MtaR, a regulator of methionine transport, is critical for survival of group B streptococcus in vivo. J Bacteriol 185:6592–6599. doi:10.1128/JB.185.22.6592-6599.2003. 14594832 PMC262094

[24] Zhang C, An H, Hu J, Li J, Zhang W, Lan X, Deng H, Zhang J-R. 2021. MetR is a molecular adaptor for pneumococcal carriage in the healthy upper airway. Mol Microbiol 116:438–458. doi:10.1111/mmi.14724. 33811693

[25] Afzal M, Shafeeq S, Kuipers OP. 2016. Methionine-mediated gene expression and characterization of the CmhR regulon in Streptococcus pneumoniae. Microb Genom 2:e000091. doi:10.1099/mgen.0.000091. 28348831 PMC5359408

[26] Hondorp ER, Matthews RG. 28 April 2006. Methionine. EcoSal Plus 2013. doi:10.1128/ecosalplus.3.6.1.7. 26443567

[27] Flores-Kim J, Dobihal GS, Fenton A, Rudner DZ, Bernhardt TG. 2019. A switch in surface polymer biogenesis triggers growth-phase-dependent and antibiotic-induced bacteriolysis. Elife 8:e44912. doi:10.7554/eLife.44912. 30964003 PMC6456293

[28] Holtje JV, Tomasz A. 1975. Lipoteichoic acid: a specific inhibitor of autolysin activity in pneumococcus. Proc Natl Acad Sci USA 72:1690–1694. doi:10.1073/pnas.72.5.1690. 239401 PMC432610

[29] Meyer L, Germain E, Maisonneuve E. 2021. Regulation of *ytfK* by cAMP-CRP contributes to SpoT-dependent accumulation of (p)ppGpp in response to carbon starvation YtfK responds to glucose exhaustion. Front Microbiol 12:775164. doi:10.3389/fmicb.2021.775164. 34803996 PMC8600398

[30] Dalebroux ZD, Swanson MS. 2012. ppGpp: magic beyond RNA polymerase. Nat Rev Microbiol 10:203–212. doi:10.1038/nrmicro2720. 22337166 PMC13198741

[31] Heuston S, Begley M, Gahan CGM, Hill C. 2012. Isoprenoid biosynthesis in bacterial pathogens. Microbiology (Reading) 158:1389–1401. doi:10.1099/mic.0.051599-0. 22466083

[32] Wilding EI, Brown JR, Bryant AP, Chalker AF, Holmes DJ, Ingraham KA, Iordanescu S, So CY, Rosenberg M, Gwynn MN. 2000. Identification, evolution, and essentiality of the mevalonate pathway for isopentenyl diphosphate biosynthesis in gram-positive cocci. J Bacteriol 182:4319–4327. doi:10.1128/JB.182.15.4319-4327.2000. 10894743 PMC101949

[33] Dewachter L, Denereaz J, Liu X, de Bakker V, Costa C, Baldry M, Sirard JC, Veening JW. 2022. Amoxicillin-resistant *Streptococcus pneumoniae* can be resensitized by targeting the mevalonate pathway as indicated by sCRilecs-seq. Elife 11:e75607. doi:10.7554/eLife.75607. 35748540 PMC9363119

[34] Willett NP, Morse GE. 1966. Long-chain fatty acid inhibition of growth of *Streptococcus agalactiae* in a chemically defined medium. J Bacteriol 91:2245–2250. doi:10.1128/jb.91.6.2245-2250.1966. 5943940 PMC316201

[35] Härtel T, Klein M, Koedel U, Rohde M, Petruschka L, Hammerschmidt S. 2011. Impact of glutamine transporters on pneumococcal fitness under infection-related conditions. Infect Immun 79:44–58. doi:10.1128/IAI.00855-10. 21078855 PMC3019899

[36] Kloosterman TG, Hendriksen WT, Bijlsma JJ, Bootsma HJ, van Hijum SA, Kok J, Hermans PW, Kuipers OP. 2006. Regulation of glutamine and glutamate metabolism by GlnR and GlnA in *Streptococcus pneumoniae*. J Biol Chem 281:25097–25109. doi:10.1074/jbc.M601661200. 16787930

[37] Gingras H, Patron K, Leprohon P, Ouellette M. 2020. Azithromycin resistance mutations in *Streptococcus pneumoniae* as revealed by a chemogenomic screen. Microb Genom 6:mgen000454. doi:10.1099/mgen.0.000454. 33074087 PMC7725334

[38] Lu P, Ma D, Chen Y, Guo Y, Chen G-Q, Deng H, Shi Y. 2013. l-Glutamine provides acid resistance for *Escherichia coli* through enzymatic release of ammonia. Cell Res 23:635–644. doi:10.1038/cr.2013.13. 23337585 PMC3641589

[39] Wang X, Zeng Y, Sheng L, Larson P, Liu X, Zou X, Wang S, Guo K, Ma C, Zhang G, Cui H, Ferguson DM, Li Y, Zhang J, Aldrich CC. 2019. A cinchona alkaloid antibiotic that appears to target ATP synthase in *Streptococcus pneumoniae*. J Med Chem 62:2305–2332. doi:10.1021/acs.jmedchem.8b01353. 30779564

[40] Russell JB, Diez-Gonzalez F. 1998. The effects of fermentation acids on bacterial growth. Adv Microb Physiol 39:205–234. doi:10.1016/s0065-2911(08)60017-x. 9328648

[41] Morlot C, Straume D, Peters K, Hegnar OA, Simon N, Villard AM, Contreras-Martel C, Leisico F, Breukink E, Gravier-Pelletier C, Le Corre L, Vollmer W, Pietrancosta N, Havarstein LS, Zapun A. 2018. Structure of the essential peptidoglycan amidotransferase MurT/GatD complex from *Streptococcus pneumoniae*. Nat Commun 9:3180. doi:10.1038/s41467-018-05602-w. 30093673 PMC6085368

[42] Vermeulen N, Ganzle MG, Vogel RF. 2007. Glutamine deamidation by cereal-associated lactic acid bacteria. J Appl Microbiol 103:1197–1205. doi:10.1111/j.1365-2672.2007.03333.x. 17897224

[43] Nguyen D, Joshi-Datar A, Lepine F, Bauerle E, Olakanmi O, Beer K, McKay G, Siehnel R, Schafhauser J, Wang Y, Britigan BE, Singh PK. 2011. Active starvation responses mediate antibiotic tolerance in biofilms and nutrient-limited bacteria. Science 334:982–986. doi:10.1126/science.1211037. 22096200 PMC4046891

[44] Li Y, Croucher NJ, Thompson CM, Trzciński K, Hanage WP, Lipsitch M. 2015. Identification of pneumococcal colonization determinants in the stringent response pathway facilitated by genomic diversity. BMC Genomics 16:369. doi:10.1186/s12864-015-1573-6. 25956132 PMC4424882

[45] Zhang R, Yang N, Ji C, Zheng J, Liang Z, Hou C-Y, Liu Y-Y, Zuo P-P. 2015. Neuroprotective effects of aceglutamide on motor function in a rat model of cerebral ischemia and reperfusion. Restor Neurol Neurosci 33:741–759. doi:10.3233/RNN-150509. 26444640

[46] Goode O, Smith A, Zarkan A, Cama J, Invergo BM, Belgami D, Cano-Muniz S, Metz J, O’Neill P, Jeffries A, Norville IH, David J, Summers D, Pagliara S. 2021. Persister *Escherichia coli* cells have a lower intracellular pH than susceptible cells but maintain their pH in response to antibiotic treatment. mBio 12:e00909-21. doi:10.1128/mBio.00909-21. 34281389 PMC8406257

[47] Bartek IL, Reichlen MJ, Honaker RW, Leistikow RL, Clambey ET, Scobey MS, Hinds AB, Born SE, Covey CR, Schurr MJ, Lenaerts AJ, Voskuil MI. 2016. Antibiotic bactericidal activity is countered by maintaining pH homeostasis in *Mycobacterium smegmatis*. mSphere 1:e00176-16. doi:10.1128/mSphere.00176-16. 27579369 PMC4999920

[48] Stokes JM, Lopatkin AJ, Lobritz MA, Collins JJ. 2019. Bacterial metabolism and antibiotic efficacy. Cell Metab 30:251–259. doi:10.1016/j.cmet.2019.06.009. 31279676 PMC6990394

[49] Orij R, Postmus J, Ter Beek A, Brul S, Smits GJ. 2009. *In vivo* measurement of cytosolic and mitochondrial pH using a pH-sensitive GFP derivative in *Saccharomyces cerevisiae* reveals a relation between intracellular pH and growth. Microbiology (Reading) 155:268–278. doi:10.1099/mic.0.022038-0. 19118367

[50] Al-Husari M, Webb SD. 2013. Regulation of tumour intracellular pH: a mathematical model examining the interplay between H^+^ and lactate. J Theor Biol 322:58–71. doi:10.1016/j.jtbi.2013.01.007. 23340437

[51] Echlin H, Frank M, Rock C, Rosch JW. 2020. Role of the pyruvate metabolic network on carbohydrate metabolism and virulence in *Streptococcus pneumoniae*. Mol Microbiol 114:536–552. doi:10.1111/mmi.14557. 32495474 PMC8538403

[52] Gaspar P, Al-Bayati FAY, Andrew PW, Neves AR, Yesilkaya H. 2014. Lactate dehydrogenase is the key enzyme for pneumococcal pyruvate metabolism and pneumococcal survival in blood. Infect Immun 82:5099–5109. doi:10.1128/IAI.02005-14. 25245810 PMC4249287

[53] Al-Bayati FAY, Kahya HFH, Damianou A, Shafeeq S, Kuipers OP, Andrew PW, Yesilkaya H. 2017. Pneumococcal galactose catabolism is controlled by multiple regulators acting on pyruvate formate lyase. Sci Rep 7:43587. doi:10.1038/srep43587. 28240278 PMC5327383

[54] El Khoury JY, Boucher N, Bergeron MG, Leprohon P, Ouellette M. 2017. Penicillin induces alterations in glutamine metabolism in *Streptococcus pneumoniae*. Sci Rep 7:14587. doi:10.1038/s41598-017-15035-y. 29109543 PMC5673960

[55] Goude R, Renaud S, Bonnassie S, Bernard T, Blanco C. 2004. Glutamine, glutamate, and alpha-glucosylglycerate are the major osmotic solutes accumulated by Erwinia chrysanthemi strain 3937. Appl Environ Microbiol 70:6535–6541. doi:10.1128/AEM.70.11.6535-6541.2004. 15528516 PMC525223

[56] Walsh RL, Camilli A. 2011. *Streptococcus pneumoniae* is desiccation tolerant and infectious upon rehydration. mBio 2:e00092-11. doi:10.1128/mBio.00092-11. 21610120 PMC3101785

[57] Vergison A, Dagan R, Arguedas A, Bonhoeffer J, Cohen R, Dhooge I, Hoberman A, Liese J, Marchisio P, Palmu AA, Ray GT, Sanders EA, Simoes EA, Uhari M, van Eldere J, Pelton SI. 2010. Otitis media and its consequences: beyond the earache. Lancet Infect Dis 10:195–203. doi:10.1016/S1473-3099(10)70012-8. 20185098

[58] Korona-Glowniak I, Mazur E, Zychowski P, Niedzielska G, Koziol-Montewka M, Malm A. 2018. Bacterial aetiology of recalcitrant acute otitis media in 62 children—high risk of pathogen colonisation after treatment. Clin Otolaryngol 43:665–669. doi:10.1111/coa.12986. 28914492

[59] Korona-Glowniak I, Zychowski P, Siwiec R, Mazur E, Niedzielska G, Malm A. 2018. Resistant *Streptococcus pneumoniae* strains in children with acute otitis media—high risk of persistent colonization after treatment. BMC Infect Dis 18:478. doi:10.1186/s12879-018-3398-9. 30253754 PMC6156860

[60] Lanie JA, Ng W-L, Kazmierczak KM, Andrzejewski TM, Davidsen TM, Wayne KJ, Tettelin H, Glass JI, Winkler ME. 2007. Genome sequence of Avery’s virulent serotype 2 strain D39 of *Streptococcus pneumoniae* and comparison with that of unencapsulated laboratory strain R6. J Bacteriol 189:38–51. doi:10.1128/JB.01148-06. 17041037 PMC1797212

[61] Lu L, Ma Y, Zhang J-R. 2006. *Streptococcus pneumoniae* recruits complement factor H through the amino terminus of CbpA. J Biol Chem 281:15464–15474. doi:10.1074/jbc.M602404200. 16597618

[62] Li J, Li J-W, Feng Z, Wang J, An H, Liu Y, Wang Y, Wang K, Zhang X, Miao Z, Liang W, Sebra R, Wang G, Wang W-C, Zhang J-R. 2016. Epigenetic switch driven by DNA inversions dictates phase variation in *Streptococcus pneumoniae*. PLoS Pathog 12:e1005762. doi:10.1371/journal.ppat.1005762. 27427949 PMC4948785

[63] Sung CK, Li H, Claverys JP, Morrison DA. 2001. An *rpsL* cassette, Janus, for gene replacement through negative selection in *Streptococcus pneumoniae*. Appl Environ Microbiol 67:5190–5196. doi:10.1128/AEM.67.11.5190-5196.2001. 11679344 PMC93289

[64] Vandal OH, Pierini LM, Schnappinger D, Nathan CF, Ehrt S. 2008. A membrane protein preserves intrabacterial pH in intraphagosomal *Mycobacterium tuberculosis*. Nat Med 14:849–854. doi:10.1038/nm.1795. 18641659 PMC2538620

[65] Miesenbock G, De Angelis DA, Rothman JE. 1998. Visualizing secretion and synaptic transmission with pH-sensitive green fluorescent proteins. Nature 394:192–195. doi:10.1038/28190. 9671304

[66] Eguchi Y, Fukumori Y, Taoka A. 2018. Measuring magnetosomal pH of the magnetotactic bacterium Magnetospirillum magneticum AMB-1 using pH-sensitive fluorescent proteins. Biosci Biotechnol Biochem 82:1243–1251. doi:10.1080/09168451.2018.1451739. 29557302

[67] Tang H, Wang X, Xu L, Ran X, Li X, Chen L, Zhao X, Deng H, Liu X. 2016. Establishment of local searching methods for orbitrap-based high throughput metabolomics analysis. Talanta 156–157:163–171. doi:10.1016/j.talanta.2016.04.051. 27260449

[68] Wang Y, Wen Z, Pan X, Briles DE, He Y, Zhang JR. 2018. Novel immunoprotective proteins of *Streptococcus pneumoniae* identified by opsonophagocytosis killing screen. Infect Immun 86:e00423-18. doi:10.1128/iai.00423-18. 29891544 PMC6105882

