# Supplementary figures and images for: Amino Acid Starvation-Induced Glutamine Accumulation Enhances Pneumococcal Survival
Source: mSphere. 2023 Apr 5;8(3):e00625-22. doi: 10.1128/msphere.00625-22 (PMC10286718; doi:10.1128/msphere.00625-22)

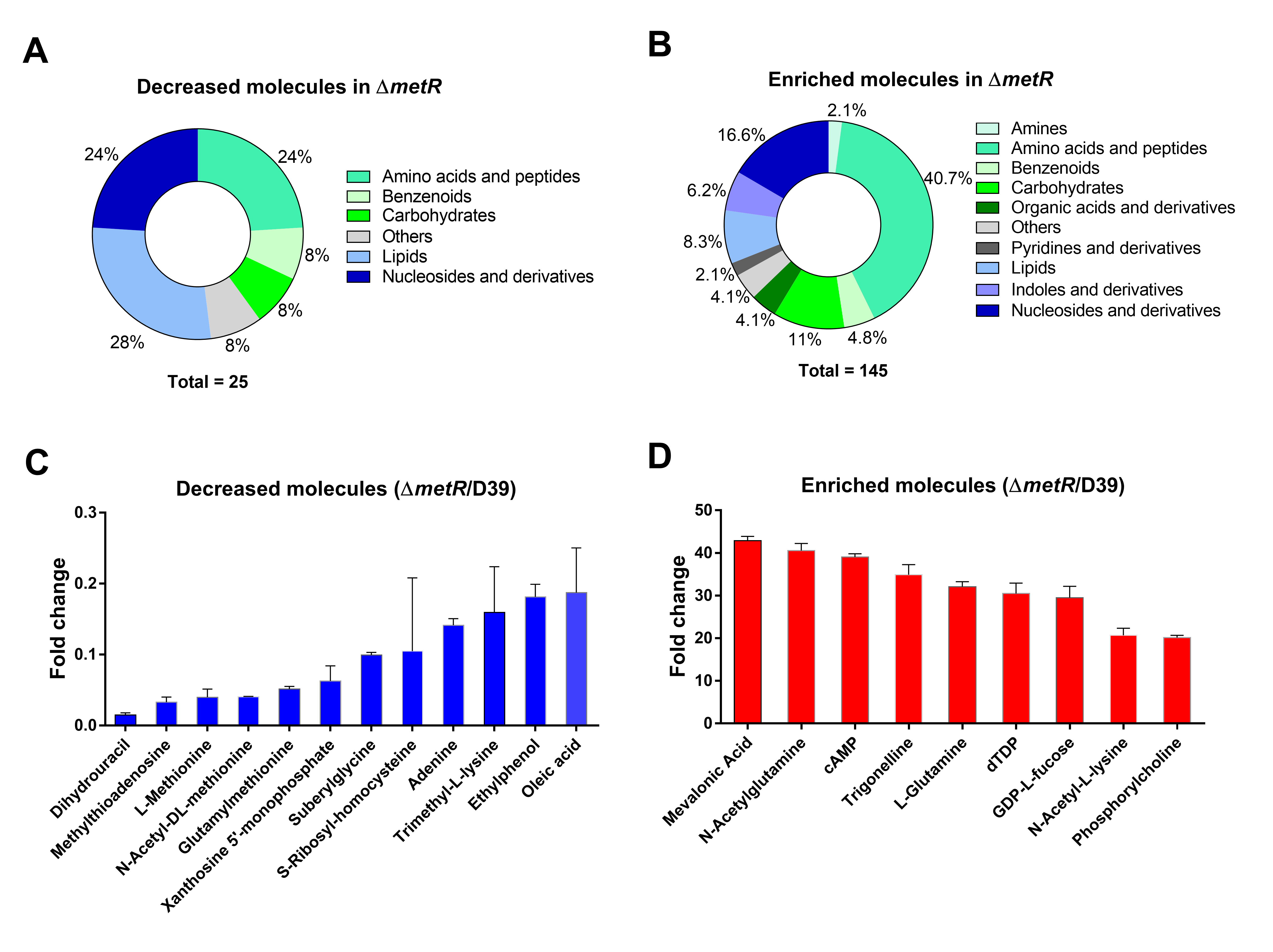

Supplement: FIG S1 [file msphere.00625-22-s0001.tif]

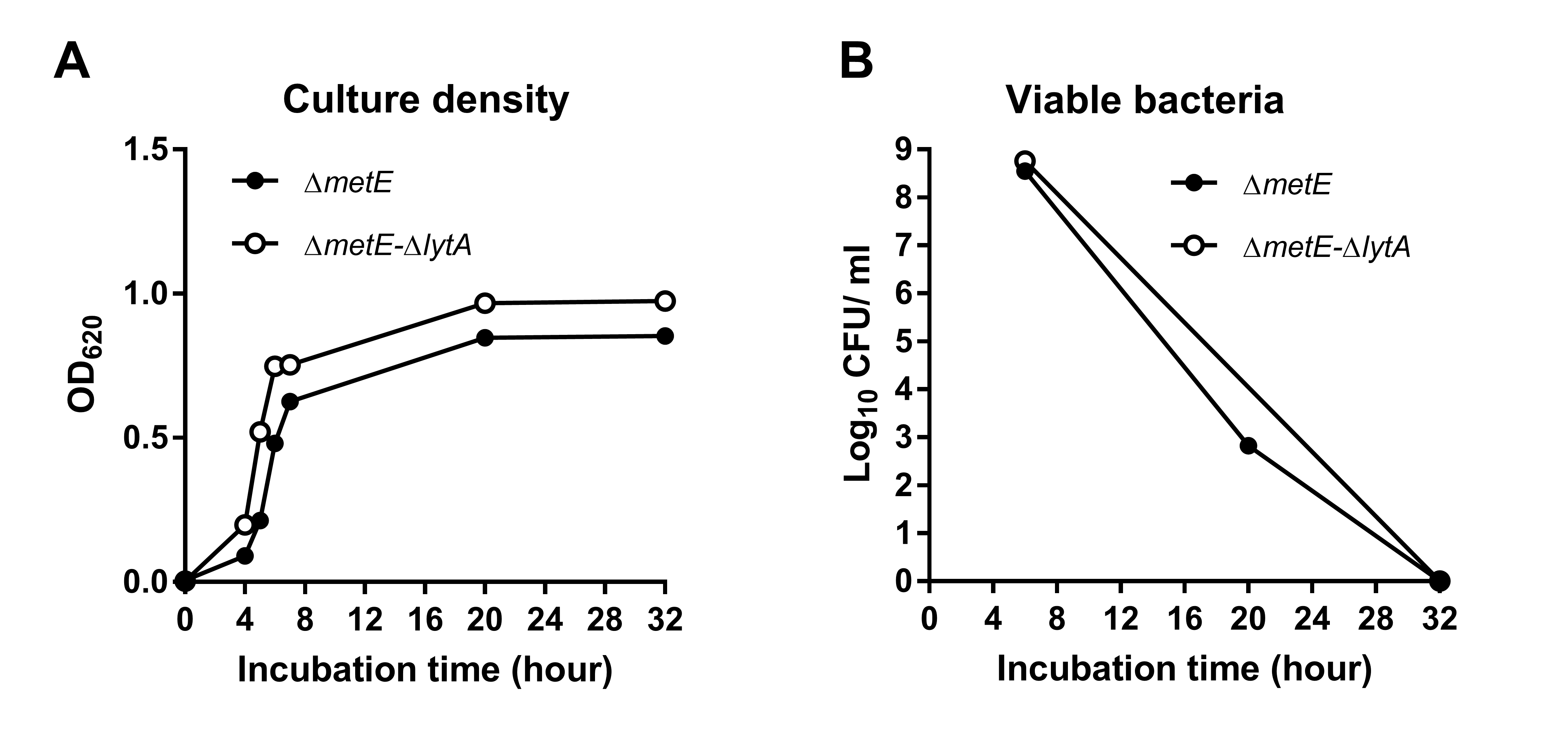

Supplement: FIG S2 [file msphere.00625-22-s0003.tif]

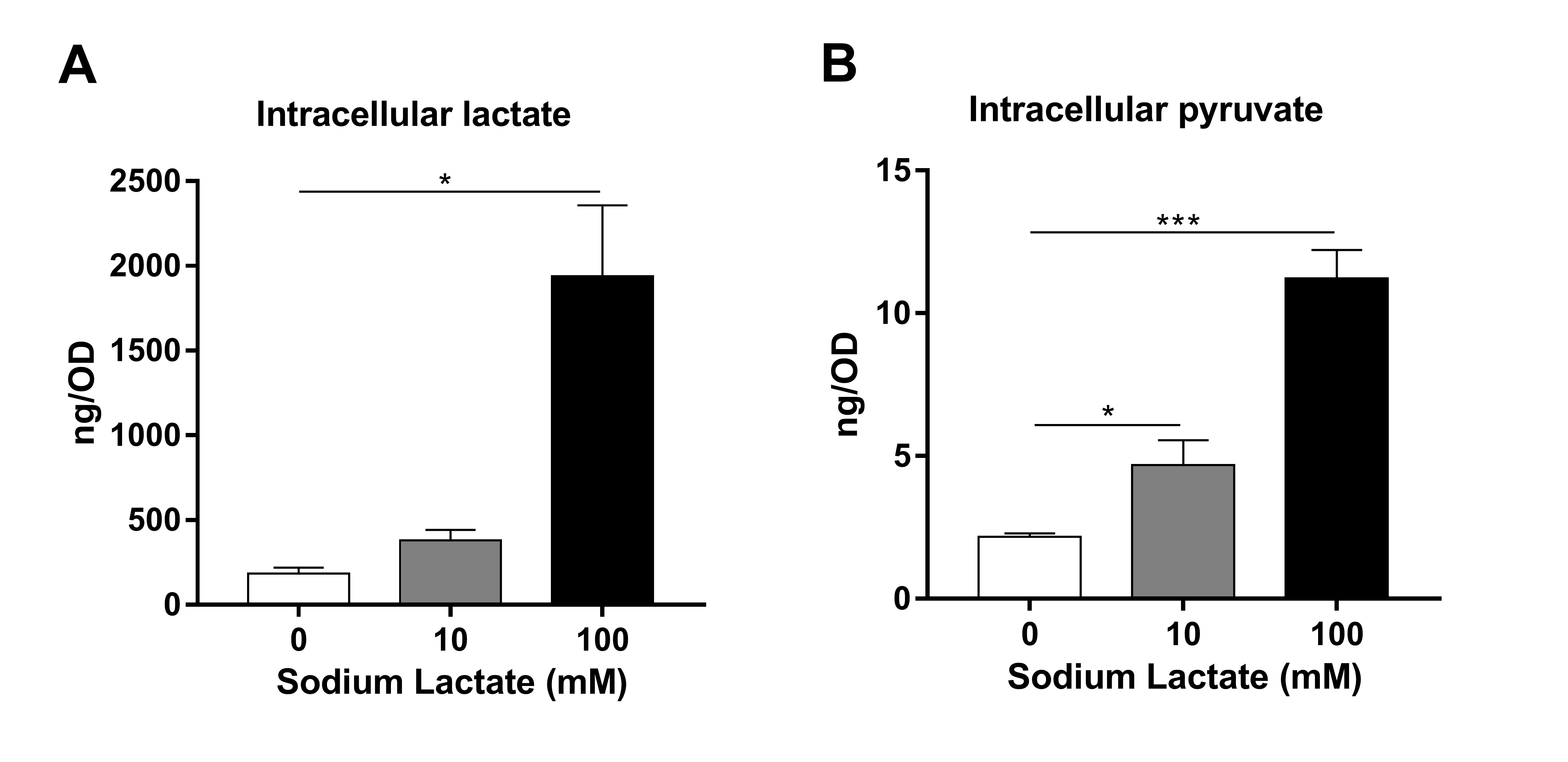

Supplement: FIG S3 [file msphere.00625-22-s0002.tif]

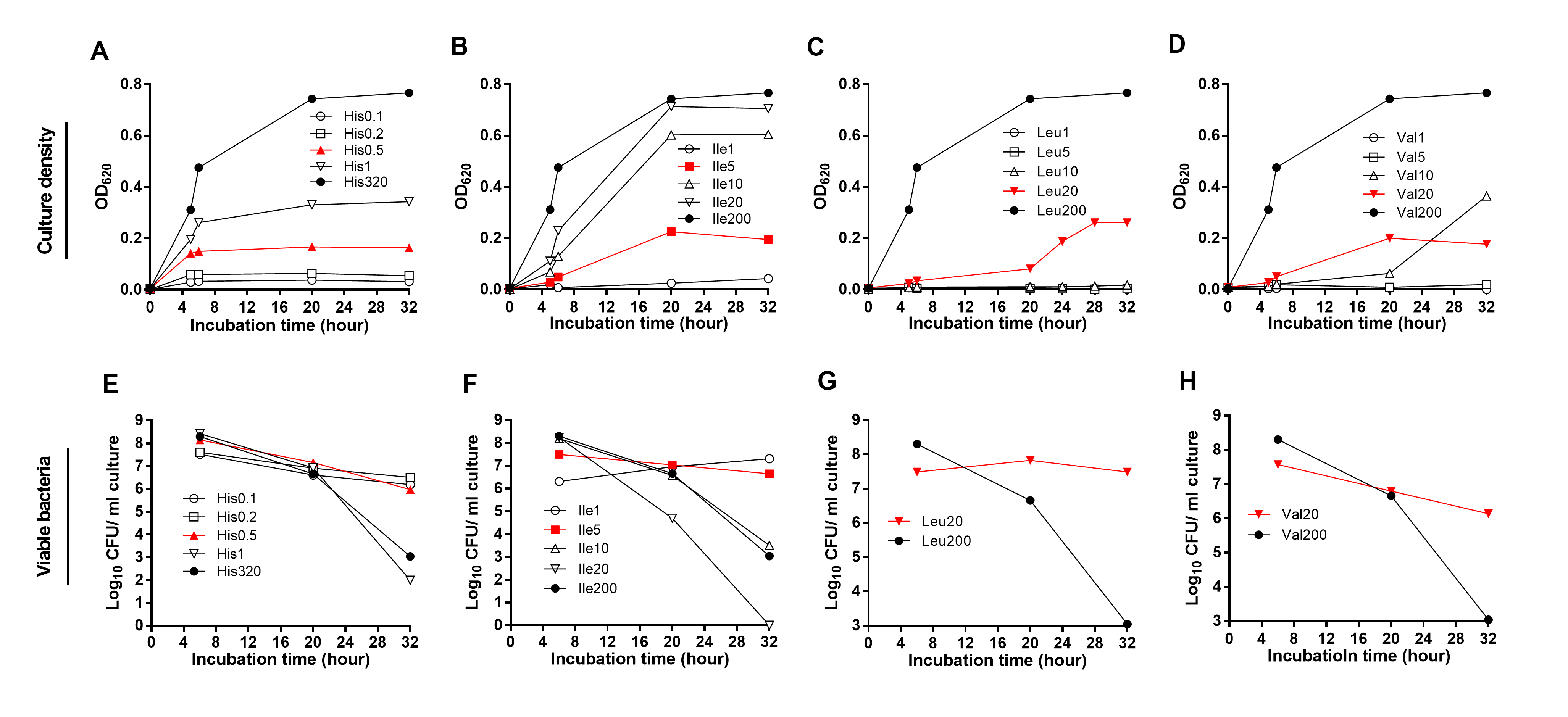

Supplement: FIG S4 [file msphere.00625-22-s0004.tif]
