# Supplementary material for: Amino Acid Starvation-Induced Glutamine Accumulation Enhances Pneumococcal Survival
Source: mSphere. 2023 Apr 5;8(3):e00625-22. doi: 10.1128/msphere.00625-22 (PMC10286718; doi:10.1128/msphere.00625-22)
Supplement: TABLE S5 [file msphere.00625-22-s0009.docx]

**Table S5. Primers used in this study**

| **Primer number** | **Sequence (5’-3’)** |
| --- | --- |
| Pr14008 | CTTCTCAGAATCTAGGAGCTAAGCG |
| Pr14009 | GAGATCTAGAATTCTACTCCTTATCAATTAAAACA |
| Pr14010 | GAGACTCGAGTAATGGAATGTCTTTCAAATCAGAA |
| Pr14011 | CCATCCTATACAGTGAAGATGGGAA |
| Pr16639 | TTTGAAAGATGTCAGCATTTCTGCC |
| Pr16640 | GAGATCTAGAATCACTCAAAATGACTTTCGTCTAA |
| Pr16641 | GAGACTCGAGCGATTGGAACATCTGGTCGTTCTTT |
| Pr16642 | CGTTAGCGTCTGCTTCAACCTTAAC |
| Pr16643 | GGTCTCGCGATCACTCAAAATGACTTTCGTCTAA |
| Pr16644 | GGTCTCGATCGATTGGAACATCTGGTCGTTCTTT |
| Pr16645 | GGAGACCTCTACCGTTTTCTATCTC |
| Pr16646 | GAGATCTAGACATAAAATGATTTTCGCTTTCTAAT |
| Pr16647 | GAGACTCGAGCTTTAGATTAAATCTGTCTTTTCAC |
| Pr16648 | AATCAATTTCCCAGAAGTAGGTTTG |
| Pr16649 | GGTCTCGAGCATAAAATGATTTTCGCTTTCTAAT |
| Pr16650 | GGTCTCGTGCTTTAGATTAAATCTGTCTTTTCAC |
| Pr16651 | GATGTGCAGAATCCTGAACAGGCTC |
| Pr16652 | GAGATCTAGACTATTTTCCTCGTTCAGCCATGAGA |
| Pr16653 | GAGACTCGAGGATTGAATTTCTCAACTTTTTTACA |
| Pr16654 | TACATGAACCAAGGAATTGACCCAG |
| Pr18409 | CAACTGGCTCACTACAATACCGAAG |
| Pr18410 | GAGATCTAGAATAAACCATTAGTTCATCTCCTTTC |
| Pr18411 | GAGACTCGAGTTTTCTTAAACAAAGGAAAATGATA |
| Pr18412 | TCAACCTTATTAGCTGCTGGAATGC |
| Pr18539 | CATGCCATGGGCAGCAGCCATCATCACCATCACCATGATGAATATTTACGCATCGGTATGG |
| Pr18540 | CGCGGATCCTTATTGTTCCTTGATCATACGATCCATC |
| Pr18541 | CATGCCATGGGCAGCAGCCATCATCACCATCACCATGATACTAGTATCGCAGACATTCAAA |
| Pr18542 | CGCGGATCCTTAATCTGCAGTATGGCTAGATGGTTTG |
| Pr18543 | CATGCCATGGGCAGCAGCCATCATCACCATCACCATGAAACGATTAAGATTGTTTCTGATA |
| Pr18544 | CGCGGATCCTTATGATGAAGCAGTCCATTTCTTGATA |
| Pr18545 | CATGCCATGGGCAGCAGCCATCATCACCATCACCATGCTATTCCTGTTAAGGCTAAATATA |
| Pr18546 | CGCGGATCCTTAGCTAGCTAGGTATTTGTCAAGAATC |
| Pr18547 | CATGCCATGGGCAGCAGCCATCATCACCATCACCATGAAACTAGTGGAGATAATTGGTCAA |

**Table S5. Primers used in this study (continued)**

| Pr18548 | CGCGGATCCTTACTGTCCTTCTTTTACTTCTTTGGTT |
| --- | --- |
| Pr18868 | GCCAAAGCAGAAACGATTAAGATTG |
| Pr18869 | GGTCTCGTAAGAAGAATCGCTGGCAATGATATAT |
| Pr18870 | GGTCTCGCTCAAACCTTCCTAACAGAAAATCAAA |
| Pr18871 | CATGGTTTTTCCATAAGAGATACCC |
| Pr18874 | GGTCTCGCTTATGCCCCTTTTGTTTTCCAAAATT |
| Pr18875 | GGTCTCGTGAGCAGCAGTTCCGTTTTTAACACCG |
