# Supplementary material for: Amino Acid Starvation-Induced Glutamine Accumulation Enhances Pneumococcal Survival
Source: mSphere. 2023 Apr 5;8(3):e00625-22. doi: 10.1128/msphere.00625-22 (PMC10286718; doi:10.1128/msphere.00625-22)
Supplement: TABLE S6 [file msphere.00625-22-s0010.docx]

**Table S6. PCR amplifications used for pneumococcal mutagenesis in this study**

| **Strain ID** | **Primers** | | | | **Digestion** | **Template DNA** | **Parent strains** |
| --- | --- | --- | --- | --- | --- | --- | --- |
|  | **Upstream** | | **Downstream** | |  |  |  |
| TH16194 | Pr18868/  Pr18869 | Pr18874/  Pr18875 | | Pr18870/  Pr18871 | BsaI/BsaI/BsaI | Synthesized | TH16191 |
| TH16216 | Pr14008/Pr14009 | | Pr14010/Pr14011 | | XbaI/XhoI | TH4306 | TH9660 |
| TH16218 | Pr16645/Pr16646 | | Pr16647/Pr16648 | | XbaI/XhoI | TH4306 | TH9660 |
| TH16219 | Pr16645/Pr16649 | | Pr16650/Pr16648 | | BsaI/BsaI | TH4306 | TH16218 |
| TH16220 | Pr16651/Pr16652 | | Pr16653/Pr16654 | | XbaI/XhoI | TH4306 | TH9660 |
| TH16221 | Pr18409/Pr18410 | | Pr18411/Pr18412 | | XbaI/XhoI | TH4306 | TH9660 |
| TH16222 | Pr16639/Pr16640 | | Pr16641/Pr16642 | | XbaI/XhoI | TH4306 | TH9660 |
| TH16223 | Pr16639/Pr16643 | | Pr16644/Pr16642 | | BsaI/BsaI | TH4306 | TH16222 |
